# Cardiometabolic Phenotypes and Dietary Patterns in Albanian University-Enrolled Young Adults: Cross-Sectional Findings from the *Nutrition Synergies* WHO-Aligned Sentinel Platform

**DOI:** 10.3390/nu17213395

**Published:** 2025-10-29

**Authors:** Vilma Gurazi, Sanije Zejnelhoxha, Megisa Sulenji, Lajza Koxha, Herga Protoduari, Kestjana Arapi, Elma Rexha, Flavia Gjata, Orgesa Spahiu, Erand Llanaj

**Affiliations:** 1 Faculty of Biotechnology and Food, Agricultural University of Tirana, Kodër-Kamëz, SH1 Highway, 1029 Tirana, Albania; zejnelhoxhas@ubt.edu.al (S.Z.); megisasulenji03@gmail.com (M.S.); protoduariherga@gmail.com (H.P.); elmarexha01@gmail.com (E.R.);; 2 Department of Public Health and Epidemiology, Faculty of Medicine, University of Debrecen, Kassai Street 26/B, H-4028 Debrecen, Hungary; 3 MTA-DE Public Health Research Group of the Hungarian Academy of Sciences, 1051 Budapest, Hungary; 4 Department of Molecular Epidemiology, German Institute of Human Nutrition, 14558 Nuthetal, Germany; 5 German Centre for Diabetes Research (DZD), 85764 München-Neuherberg, Germany; 6 Institute for Health Metrics and Evaluation (IHME Collaborator), University of Washington, Seattle, WA 95108, USA

**Keywords:** Albania, youth, cardiometabolic risk, diet quality, saturated fat, polyunsaturated fat, surveillance, Western Balkans, nutrition transition

## Abstract

**Background**: Albania is undergoing rapid nutrition transition, yet cardiometabolic (CM) risk in young adults is poorly characterized. We report baseline, cross-sectional findings from a WHO-aligned sentinel study examining diet, physical activity and early CM phenotypes, with fat quality examined as a modifiable exposure. **Methods**: Young adults recruited on campus (*n* = 262; median age, 21 years; 172 women, 90 men) underwent standardized anthropometry, seated blood pressure (BP) and fasting glucose (FG). Diet was assessed by two interviewer-administered 24 h recalls and activity outlined by the IPAQ-short form. We derived potential renal acid load (PRAL) and a MASLD-oriented nutrient score, computed a composite CM risk score (cCMRS: sex-standardized mean of WHtR, mean arterial pressure, FG) and fitted prespecified energy-partition models for isocaloric +5% of energy substitutions (SFA → PUFA; SFA → MUFA) with Benjamini–Hochberg false discovery rate (FDR) control. **Results**: Despite normal average BMI (23.4), risk clustering was common: elevated BP in 63% of men and 30% of women, impaired FG (100–125 mg/dL) in almost one third and central adiposity (WHtR ≥ 0.5) in 51% of men and 24% of women. Diets were SFA-rich (~17–19%E), sodium-dense and low in fiber and several micronutrients (e.g., vitamin D, folate, potassium). In isocaloric models, SFA → PUFA was associated with more favorable nutrient signatures: MASLD-oriented score −28% (*p* < 0.001; FDR-significant) and PRAL −33% (*p* = 0.007; FDR-borderline/suggestive). **Conclusions**: A waist-centric CM subphenotype—central adiposity co-occurring with upward BP shifts and intermittent dysglycemia—was detectable in young adults despite normal average BMI, against a background of poor diet quality and low activity. These baseline surveillance signals are not causal effects. Integration into routine with WHO-aligned NCD surveillance is feasible. Prospective follow-up (biomarker calibration, device-based activity, repeated waves) will refine inferences and inform scalable proactive prevention.

## 1. Introduction

Malnutrition in all its forms is rising globally and accelerating cardiometabolic diseases (CMDs), particularly where traditional diets are giving way to energy-dense, nutrient-poor, ultra-processed patterns [1,2]. Albania exemplifies this transition. Noncommunicable diseases now account for roughly two in three deaths (≈22,500 annually) [3,4], while more than 60% of adults have excess body weight (≈25% obesity) and around one in ten lives with type 2 diabetes (T2D) [5,6]. Hypertension affects nearly one in two adults aged 30–79, yet only about 8% achieve blood-pressure (BP) control, pointing to a large reservoir of hidden hypertension [7]. Likewise, surveys indicate that up to one-third of adults with diabetes remain undiagnosed, underscoring a substantial burden of unrecognized cardiometabolic (CM) risks [8,9].

Dietary exposures compound these vulnerabilities. In 2020, Albania ranked first globally for sugar-sweetened beverage-attributable disease burden, corresponding to approximately 1560 new T2D and 1950 CVD cases per million adults each year [10]. Mean salt intake is ≈13 g/day—more than twice the WHO target (<5 g/day)—while progress to diet-related NCDs remains limited [11,12]. Among younger groups, over one in three children are overweight by age 8–9 [13] and most adolescents fail to meet physical-activity recommendations (insufficient physical activity: 76% of boys, 86% of girls) [14], suggesting that risk trajectories are seeded early and reinforced through adolescence.

The European Commission’s consecutive 2022–2024 country accession reports have consistently identified nutrition in Albania as being in a critical state, citing rising obesity rates, limited access to affordable healthy foods and insufficiently developed preventive health services [15,16,17]. These findings highlight an urgent need for reproducible, context-specific evidence to inform youth-focused interventions. Yet young adults remain largely absent from national monitoring systems: school-age surveys dominate, screening seldom includes those under 35 years and dietary surveillance does not quantify nutrient intake or align with WHO and STROBE-nut standards [3,5,18].

To address this gap, we established the Nutrition Synergies (NutriSYN) Sentinel Study, a WHO-aligned, cross-sectional baseline among Albanian university students, designed to yield reproducible, internationally comparable indicators of diet quality and subclinical CM phenotypes.

Using harmonized procedures, we combined interviewer-administered 24 h dietary recalls and a brief physical-activity instrument with standardized phenotyping (anthropometry, BP, fasting glycemia). Dietary exposures were summarized through pathway-oriented indices—including dietary renal acid load (PRAL) [19], a nutrient score oriented to metabolic dysfunction-associated steatotic liver disease (MASLD) risk [19], adherence to WCRF/AICR cancer-prevention recommendations [20] and a modified Life’s Essential 8 (LE8) [21]—and CM risk was captured through a composite endophenotype (derived from waist-to-height ratio (WHtR), BP and fasting glucose (FG).

CM risks often emerge early in life, shaped by cumulative lifestyle exposures and the rapid nutrition transition. In Albania, this transition has produced a dual landscape—persisting undernutrition coexisting with rising overweight and metabolic disturbances—yielding mixed risk typologies among youth. Within this context, we define the *waist-centered cardiometabolic subphenotype* as the co-occurrence of central adiposity, elevated BP, and impaired fasting glycemia, even among individuals of normal weight. This subphenotype represents a sentinel molecular–metabolic signal of early vascular strain and impaired metabolic flexibility, marking a shift from adaptive to maladaptive energy partitioning before overt disease manifests. It matters beyond descriptive epidemiology because it captures the earliest convergence of modifiable dietary exposures, subclinical metabolic dysfunction, and CM risk clustering in youth—a clinically actionable stage for prevention. Building on regional and WHO surveillance evidence, we hypothesized that such waist-centered risk constellations are already detectable among young adults, and that differences in dietary fat quality and overall nutrient balance would differentiate these emerging metabolic patterns.

Within this sentinel framework, we characterized the magnitude and distribution of early cardiometabolic tendencies and examined their associations with prespecified dietary exposures, including isocaloric substitution models (SFA → PUFA and SFA → MUFA). These hypothesis-generating analyses establish a transparent baseline for ongoing monitoring and policy evaluation. The *NutriSYN* platform is designed for longitudinal follow-up incorporating biomarker calibration, accelerometry and multi-omic profiling to refine etiologic inference and track the real-world impact of upstream interventions. While causal inference is not implied, this study demonstrates the feasibility and clinical relevance of WHO-aligned sentinel surveillance as a molecular epidemiologic early-warning system with evidence directly applicable to clinicians, public-health practitioners and policy stakeholders committed to proactive, scalable prevention.

## 2. Materials and Methods

### 2.1. Study Design and Setting

We conducted a cross-sectional, baseline investigation within a WHO-aligned sentinel platform for youth cardiometabolic surveillance in Albania (Nutrition Synergies, NutriSYN) between November 2024–March 2025. The source population was university students recruited via on-campus clinics scheduled on predefined days under a pragmatic sampling framework [22,23]. Field procedures followed harmonized SOPs, calibrated devices and duplicate measurements for anthropometry and BP to ensure QC/QA and intra-observer reliability (Supplementary Material) [24]. All interviewers were nutrition graduates trained on the Multiple-Pass Method; 10% of recalls were double-checked for quality. Inter-rater agreement was high (ICC ≥ 0.90), with discrepancies adjudicated by a senior nutritionist (SZ). Field interviewers were not financially compensated and contributed voluntarily following study SOPs and quality-assurance procedures.

### 2.2. Participants and Recruitment

**Figure 1:**
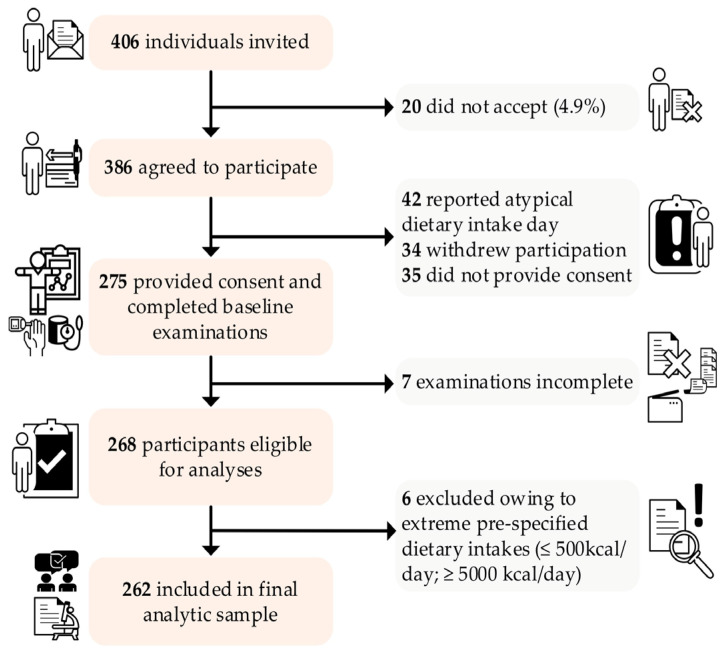
Flow of participants through the study.

Recruitment used institutional announcements and staffed assessment clinics. Eligibility (pre-specified): capacity for informed consent, age within study bounds, protocol fasting, no acute intercurrent illness and not pregnant. Screening logs documented eligibility, reasons for non-participation and protocol deviations. A STROBE flow diagram (Figure 1) summarizes screening, enrollment and derivation of the analytic sample. In line with our exposure-oriented sentinel design, no post hoc exclusions were made based on measured outcomes.

### 2.3. Anthropometrics, BP and Glycemia

Height and weight were measured with calibrated stadiometers and scales; BMI was calculated as kg/m^2^. Waist and hip circumferences were measured at standard landmarks; WHtR was computed as waist (cm)/height (cm). Central obesity used IDF Europid cut-points (waist ≥ 94 cm men; ≥80 cm women) [25,26]. BP was obtained by trained staff using standardized protocol with appropriate cuffs and duplicate readings; categories followed ESC/ESH thresholds (highest of SBP/DBP) [27]. Fasting glycemia was measured as FG after ≥8 h fast; descriptive categories used clinical cut-points (normal < 100 mg/dL; impaired 100–125 mg/dL; provisional diabetes ≥ 126 mg/dL on a single measurement) [28]. Participants with FG ≥ 126 mg/dL were notified and advised to seek clinical follow-up.

### 2.4. Dietary Assessment and Lifestyle

Habitual intake was assessed using two interviewer-administered, non-consecutive 24 h recalls (Multiple-Pass Method) conducted under SOPs with real-time plausibility checks [29,30]. Portion size estimation used a culturally adapted pictorial atlas and local household-measure conversions. Nutrient derivation used NutriSurvey with a custom Albanian food composition database (adapted from prior work [30]) cross-checked against McCance and Widdowson to harmonize energy and nutrient values [31]. Energy adjustment followed nutrition epidemiology conventions (e.g., macronutrients as % energy; fiber g/1000 kcal) [32]. Physical activity was assessed by the IPAQ-Short Form (forward–back translated/culturally adapted) [33,34,35].

### 2.5. Diet Quality and Composite Cardiometabolic Risks Indices

To capture multidimensional diet–phenotype relationships, we applied a series of pathway-oriented indices designed to reflect mechanistic domains relevant to cardiometabolic health. The dietary renal acid load (PRAL) [19] was computed as an indicator of acid–base balance and renal–metabolic strain, providing a nutrient-based estimate of systemic acid load. Hepatic–metabolic quality was assessed using a MASLD-oriented nutrient score [20] constructed a priori from nutrients implicated in hepatic steatosis, where higher values reflect a less favorable profile.

Overall diet quality and behavioral alignment with chronic-disease prevention were evaluated through the 2018 WCRF/AICR adherence score [36], incorporating seven applicable components—healthy weight, physical activity, a plant-forward dietary pattern, limited consumption of energy-dense ultra-processed foods, red and processed meat, sugar-sweetened beverages and alcohol. We further derived a modified six-component Life’s Essential 8 (LE8) score, encompassing diet, physical activity, BMI, BP, FG and smoking (used in place of the nicotine-exposure metric) [21]. Each component was scored on the standard AHA 0–100 scale, with the overall LE8 expressed as the mean of component scores to represent integrated cardiometabolic health.

To summarize early cardiometabolic susceptibility, we computed a composite cardiometabolic risk score (cCMRS) [37], calculated as the mean of sex-standardized z-scores for WHtR, mean arterial pressure (MAP) and FG, where higher values indicate greater risk. Detailed algorithms, weighting schemes and specifications of omitted LE8 metrics are provided in the Supplementary Material.

### 2.6. Energy Intake–Expenditure Plausibility (Goldberg)

Total energy expenditure (TEE) was estimated from predictive equations [38,39,40,41,42] multiplied by activity level (IPAQ-derived). Total energy intake (TEI) to TEE ratios were examined using Goldberg categories descriptively (low/concordant/high); no exclusions were made to avoid selection bias. Visualizations include sex-specific TEI–TEE scatterplots with OLS fits and 95% CIs and a Sankey linking sex to Goldberg categories (Figure S1).

### 2.7. Statistical Analysis

**Figure 2:**
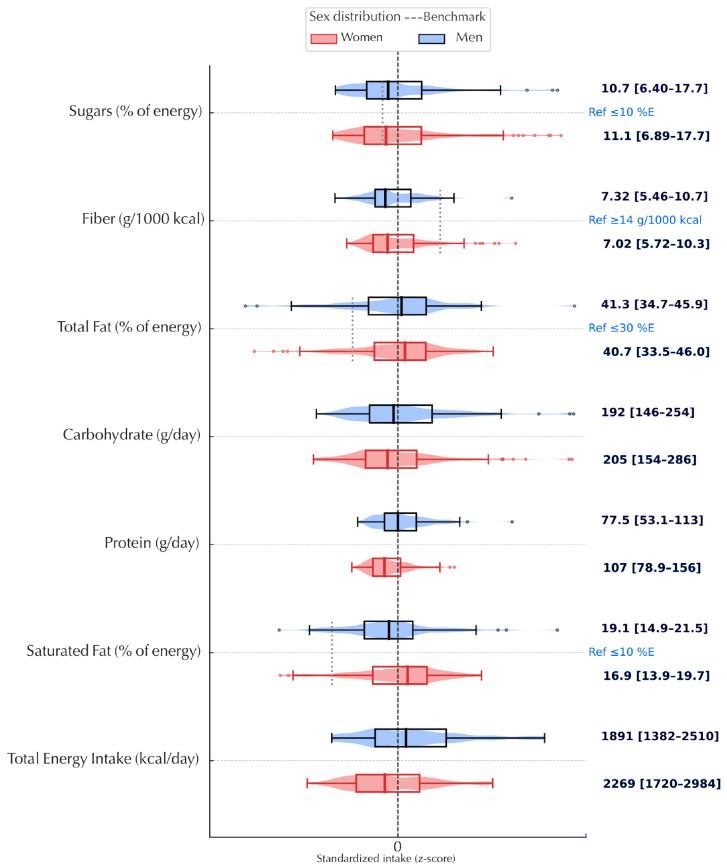
Distribution of energy and macronutrient intake by sex. *Note*: Violin plots show sex-specific distributions; interior boxes indicate the median (center line) and interquartile range (IQR). Vertical reference lines benchmarks: SFA 10%E, total fat 30%E, fiber 14 g/1000 kcal and sugars 10%E. “Standardized intake (z-score)” denotes the horizontal scaling used for plotting; interpretation relies on the raw-unit labels and benchmark lines.

**Figure 3:**
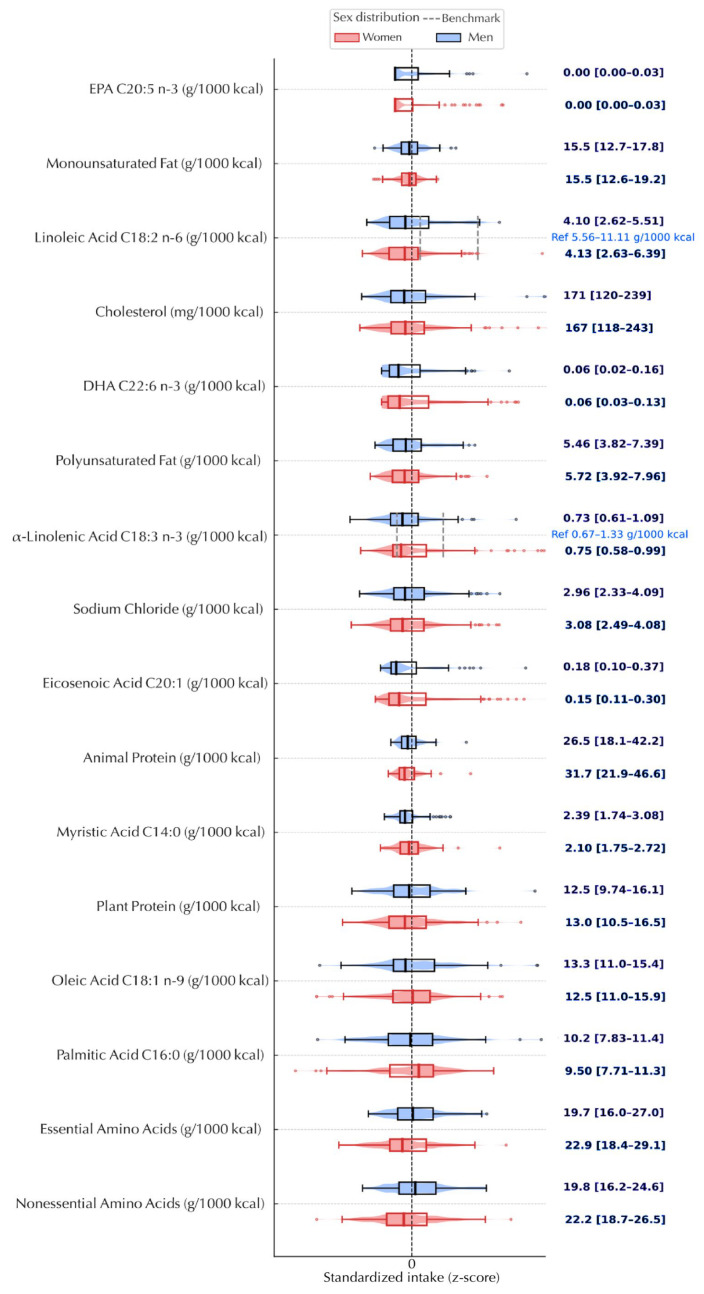
Specific fats, protein sources and selected nutrient densities, by sex. *Note*: Violin plots show sex-specific distributions with medians (center line) and IQRs. The x-axis uses standardized intake (z-score), but raw units (g/1000 kcal or mg/1000 kcal) are referenced. Vertical bands indicate recommended ranges for linoleic acid (5–10%E)/α-linolenic acid (0.6–1.2%E). Values of 0 for EPA/DHA reflect non-consumption. (1 g salt ≈ 400 mg sodium).

**Figure 4:**
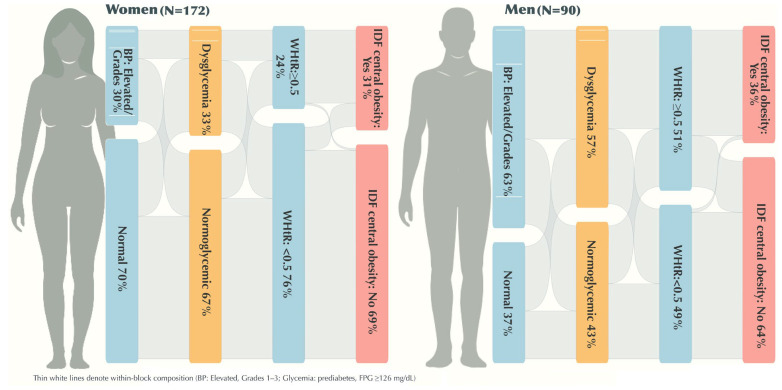
Sex-specific cardiometabolic risk and participant allocation across sequential categories. *Note*: Flows depict sequential clustering of cardiometabolic risk in Albanian university students (*n* = 262). Blood pressure (ESC/ESH 2018): Optimal <120/<80 mmHg, normal 120–129/80–84, high-normal 130–139/85–89, grade 1 140–159/90–99, grade 2 160–179/100–109, grade 3 ≥180/≥110. Glycemia (ADA 2023): Normoglycemia <100 mg/dL, impaired fasting glucose 100–125, provisional diabetes ≥ 126. Central adiposity (IDF Europid): Waist ≥ 94 cm (men), ≥80 cm (women). WHtR: Dichotomized at 0.5.

Continuous variables are summarized as medians (IQR) and categorical variables as counts (row %), stratified by sex. We assessed distributional shape with Shapiro–Wilk and visual diagnostics (histograms, Q–Q plots) and means (SD) are reported where approximately normal. We also show a complete panel of variable correlations for transparency (Figure S1). Nutrient panels were z-standardized for cross-nutrient comparability and displayed as violin plots with embedded boxplots; reference benchmarks are indicated on panels and in legends (Figure 2, Figure 3, Figures S3 and S4). A Sankey diagram summarizes co-occurrence across BP, central adiposity and glycemic strata (Figure 4). Diet–phenotype associations used multivariable energy-partition linear models with macronutrients as % energy (E%).

All energy-yielding macronutrients entered simultaneously; carbohydrate served as the reference so that each coefficient represents a 1 E% substitution of the indexed macronutrient replacing carbohydrate. Pre-specified contrasts were +5 E% reallocations for SFA → PUFA and SFA → MUFA, reported as follows: (i) SBP and FG: identity link (mmHg; mg/dL); (ii) PRAL, MASLD-oriented score, WHtR: log-transformed with back-transformed % differences and (iii) LE8, WCRF, cCMRS: SD change per +5 E% substitution (higher LE8/WCRF = more favorable; higher cCMRS = higher risk). Adjustment sets (a priori) included age, sex, current smoking and continuous physical activity; macronutrient E% were mean-centered. Inference used HC3 standard errors; VIF < 5, residual and influence checks (Cook’s distance) showed no material violations. Multiplicity was controlled within the primary family (PRAL, MASLD-oriented score) using Benjamini–Hochberg FDR (q < 0.05) [43,44]; other outcomes were secondary/exploratory. Analyses used complete-case datasets with exact denominators in legends; no imputation was performed for primary results. A schematic representation of the isocaloric substitution framework and covariate structure is provided in Scheme S1 (Supplementary Methods) to support reproducibility. An additional comprehensive conceptual overview of the *NutriSYN* framework, showing how PRAL, MASLD-oriented, WCRF/AICR and LE8 indices relate to the composite cCMRS), is provided in Supplementary Figure S5. Data management and modeling used IBM SPSS v21, Python 3.13 (pandas, numpy, statsmodels, matplotlib) and R 4.5. Reporting adheres to STROBE-nut [45] (Supplementary Material). Extended protocols, device specifications, QC details (including duplicate measurement procedures), database builds and full scoring algorithms are provided in Supplementary Methods.

## 3. Results

### 3.1. Participant Characteristics and Sample

Of 406 students invited, 386 (95.1%) expressed interest (Figure 1). Before standardized examination, 111 of those interested (28.8%) did not proceed (non-typical intake day, voluntary withdrawal, or no written consent), yielding 275 examined (67.7% of invitees). Seven examined participants lacked a core measure (*n* = 268 eligible); six additional participants were excluded per prespecified dietary-intake thresholds (≤500 or ≥5000 kcal/day), resulting in a final analytic sample of 262 students (64.5% of invitees; 172 women [65.6%], 90 men [34.4%]). Median age was 21 years in both sexes (Table 1). Mean BMI was in the normal range (≈22.5–23.4 kg/m^2^), yet central adiposity diverged by sex. Median waist circumference was 88.2 cm in men versus 75.0 cm in women; median waist-to-height ratio (WHtR) was 0.51 versus 0.46, respectively. A WHtR ≥ 0.5 (i.e., waist at least half of height) occurred in 51% of men and 24% of women, indicating a substantial waist-centric phenotype despite normal BMI distributions. By IDF cut-points, central obesity was present in 31% of women and 36% of men. BP distributions were right-shifted in men relative to women (median systolic 120 vs. 109 mmHg; diastolic 80 vs. 70 mmHg). Fasting glycemia was higher in men (median 102 mg/dL) than in women (95.2 mg/dL), placing the male median near the impaired fasting range and suggesting a clustering of modestly adverse cardiometabolic signals alongside central adiposity.

Estimated physical activity energy expenditure was higher in men (median ~3542 kcal/day) than in women (~1743 kcal/day), a contrast compatible with both body-mass and activity-pattern differences. Despite this, daily sitting time approximated 7.5–8 h in both sexes, indicating a sizeable sedentary component. Diet-quality metrics showed sex-specific contrasts: men exhibited higher potential renal acid load (PRAL; median ~33 mEq/day vs. ~14 mEq/day in women), a pattern consistent with more acidogenic intake (e.g., animal protein, refined grains) and fewer base-forming foods (e.g., vegetables, legumes, fruit). Women showed slightly higher alignment with guideline-oriented indices (WCRF and LE8). Reported alcohol intake was low overall; approximately 30–36% reported any use, with small mean intakes and heavier use confined to upper tails. Taken together, findings point to a waist-centric, mildly adverse cardiometabolic profile—more pronounced in men—characterized by higher central adiposity, right-shifted blood pressure and fasting glycemia near impairment thresholds, occurring alongside substantial sedentary time and poor diet. Signals should be interpreted descriptively, but as priority targets for surveillance and risk communication in this population.

Across both sexes, usual intake profiles converged on an energy-dense, nutrient-poor pattern. Median total fat contributed ~41% of energy (guideline ≈ 30%) and saturated fat accounted for 17–19% of energy (guideline < 10%), indicating a markedly unfavorable fat-quality ratio. Fiber density averaged ~7 g per 1000 kcal (reference, 14 g per 1000 kcal) and free sugars contributed ~11% of energy (benchmark < 10%; <5% for additional benefit). Intakes of plant-derived polyunsaturated fats were suboptimal: linoleic acid (*n*-6) centered below its benchmark, α-linolenic acid (ALA, plant *n*-3) clustered at its lower bound and long-chain marine *n*-3 fatty acids (EPA/DHA) showed floor effects, consistent with low marine-food consumption.

Cholesterol density averaged ~170 mg per 1000 kcal. Median sodium chloride density was ~5–6 g per 1000 kcal, with wide dispersion ( Figure 2; Figure 3). To contextualize magnitudes, these densities translate, for a 2000 kcal diet, to approximately 91 g total fat/day (≈1.4 × the guideline), 38–42 g saturated fat/day (≈1.7–1.9 × the upper limit), ~14 g fiber/day (≈50% of the reference), ~55 g free sugars/day (slightly above <50 g; >2 × the <25 g “stronger benefit” benchmark), ~340 mg cholesterol/day and ~10–12 g salt/day (≈2–2.4 × the WHO target of <5 g/day; ~2 teaspoons). These conversions are provided to aid interpretation and are not prescriptive. Patterns were notable for co-occurrence rather than isolated deviations.

First, fat quality skewed toward saturated fat while signals for plant-based PUFA (*n*-6, ALA) were low and EPA/DHA frequently near zero—an imbalance consistent with limited use of plant oils, nuts/seeds and oily fish. Second, carbohydrate quality showed a “high-sugar/low-fiber” profile, with free sugars modestly above threshold and fiber at half the density reference—compatible with refined-grain and sugary-beverage/snack contributions and under-representation of legumes, whole grains, vegetables and fruit. Third, sodium exposure was elevated with broad variance, suggesting that a sizable subset likely exceeds the already high median, a pattern typically driven by processed and restaurant foods rather than discretionary salt alone. Cholesterol density tracked with the observed fat pattern; although contemporary guidance prioritizes overall fat quality over dietary cholesterol per se, both metrics tended to move in tandem.

The size of these gaps is clinically meaningful at the dietary-pattern level: saturated fat and salt were approximately doubled relative to targets, while fiber was halved. By contrast, free sugars sat nearer the threshold (median ~11%E), implying that small, sustained reductions could return many individuals below the <10%E benchmark, whereas fiber, fat quality, marine *n*-3 intake and salt will likely require more material shifts in food choices. These signals were broadly similar in men and women at the median, with sodium exhibiting the greatest dispersion and EPA/DHA the most pronounced floor effect.

Given the cross-sectional design, these findings are descriptive and should not be interpreted causally. They identify a compact set of modifiable levers for surveillance and pragmatic counseling—improving fat quality (swap animal fats for plant oils, nuts/seeds; include oily fish), raising fiber density (legumes, whole grains, vegetables, fruit), dialing down free sugars (especially beverages and confectionery) and curbing salt (processed meats, cheeses, savory snacks and restaurant items)—that align with established prevention targets. Patterns were unchanged in sensitivity analyses stratifying by Goldberg plausibility. Goldberg categories suggested sex-differential reporting (possible over-reporting in women, under-reporting in men); no exclusions were made.

Micronutrient shortfalls were common (Figures S3 and S4). Vitamin D clustered around ~1.1 µg/day; vitamin E around 7–8 mg/day; folate medians 162–189 µg/day—all below reference. For minerals, potassium medians were ~2186 mg/day (men) and ~2554 mg/day (women) (reference 3510 mg/day); calcium ~800–903 mg/day (ref 1000 mg/day); iodine ~59–61 µg/day (ref 150 µg/day); and magnesium below sex-specific references. Sodium exceeded the WHO target (2000 mg/day) in both sexes. Several other micronutrients were generally adequate (e.g., B12, vitamin A, riboflavin, vitamin C), with sex-specific nuances (Figures S3 and S4 in Supplementary Material).

### 3.2. Phenotypes of Cardiometabolic Risks

Figure 4 shows sex-specific distributions for BP, fasting glycemia and waist indices. Among women, 70% were normotensive and 30% were in elevated/grade 1–3 categories; among men, 37% normotensive and 63% elevated/grade 1–3. Normoglycemia occurred in 67% of women vs. 43% of men; impaired fasting glycemia in 33% and 57%, respectively; single-occasion FG ≥ 126 mg/dL was infrequent. WHtR ≥ 0.5 affected 24% of women and 51% of men. Sankey flows indicated that elevated BP and dysglycemia often co-occurred with central adiposity (WHtR ≥ 0.5 and IDF criteria) in both sexes. Taken together, these distributions delineate a waist-centric CM subphenotype—central adiposity, upward BP shift and intermittent glycemic elevation—that is not captured by BMI alone and is detectable in early adulthood.

### 3.3. Diet–Phenotype Associations (Isocaloric Substitution)

Given high SFA and low PUFA/fiber, we examined pre-specified isocaloric substitutions using energy-partition models (Table 2). Reallocating +5%E from SFA to PUFA was associated with a −32.8% change in PRAL (95% CI −56.6 to −9.1; q = 0.054, suggestive) and −28.2% in the MASLD-oriented nutrient score (95% CI −39.0 to −17.4; q < 0.001), indicating a shift toward more favorable nutrient signatures. SFA → MUFA showed smaller, less consistent associations (PRAL −20.9%, 95% CI −42.7 to +0.8; q = 0.238; MASLD −12.4%, 95% CI −22.3 to −2.6; q = 0.070). No statistically significant substitution effects were observed for SBP, FG or WHtR, consistent with the young age, narrow variance and cross-sectional design. Food-source context may explain the weaker MUFA signal: in this setting, MUFAs frequently co-travel with SFAs in mixed animal-based dishes (e.g., meat, dairy), whereas PUFA increases more often reflect plant-oil and plant-protein shifts that alter the broader nutrient pattern (e.g., minerals linked to PRAL). All substitution estimates are exploratory, FDR-controlled within the primary family (PRAL, MASLD score) and intended as surveillance signals rather than clinical effect sizes.

## 4. Discussion

In this WHO-aligned sentinel baseline of Albanian university students, we identified a waist-centric cardiometabolic subphenotype detectable despite generally normal mean BMI: central adiposity co-occurred with upward BP shifts and impaired fasting glycemia, alongside sub-guideline physical activity and dietary profiles characterized by high total fat/SFA, low fiber, elevated sodium and several micronutrient shortfalls. The alignments suggest that CM vulnerability in early adulthood can be visible to surveillance before overt disease and that BMI alone is insufficient for risk appraisal in this age group [46]. These results delineate the internal pattern of risk before translating to policy relevance.

Patterns observed here are consistent with signals reported in the Western Balkans and Southern Europe—low fruit/vegetable intake, high free sugars, insufficient activity and rising early CM abnormalities—while adding youth-specific, nutrient-resolved baselines for Albania [47,48,49]. The clustering we observed accords with the “metabolically at-risk normal weight” construct, in which vascular and glycemic perturbations are evident before diagnostic thresholds [50,51]. The literature indicates that sustained exposure to clustered metabolic syndrome components accelerates progression to T2D more steeply than isolated glycemic abnormalities, supporting the interpretation of these patterns as an early population-level signal [52]. Risk was unevenly distributed, concentrating in women and younger adults [53,54].

Biologically, the combination of visceral adiposity with excess saturated fat intake and low micronutrient density provides a plausible mechanistic pathway through insulin resistance and hepatic steatosis [55]. These findings can be interpreted as a population signal of emerging prehypertensive and prediabetic states and the presence of elevated BP, impaired glycemia and WHtR ≥ 0.5 in a substantial fraction of men—and a non-trivial fraction of women—reinforces the value of waist-based indices and BP measurement for routine, low-cost screening on campuses and in primary care. Environmental and behavioral exposures appear to compound these signals. Prolonged sedentary time, particularly sitting, which was high in this cohort, as well as diets enriched in processed foods, are well-established contributors to obesity and youth-onset T2D [56,57]. In this sample, usual dietary intakes diverged substantially from WHO and IOM benchmarks, with multiple micronutrients consistently below recommended levels, reinforcing a profile of nutritional inadequacy [58,59]. While these findings warrant replication, such dietary patterns are recognized drivers of CVDs and MASLD in adults, suggesting that preventive intervention during youth may be warranted [60].

In our surveillance-oriented, hypothesis-generating analyses, pre-specified isocaloric substitution models indicated that reallocating +5% of energy from SFA to PUFA was associated with more favorable nutrient signatures—lower PRAL (dietary acid load) and a more favorable MASLD-oriented nutrient score. Consistent with this signal, a lipidomics-derived multilipid score capturing SFA → unsaturated fat replacement predicted lower CVD (−32%; 95% CI −42 to −21) and type 2 diabetes (−26%; 95% CI −35 to −15) in EPIC-Potsdam, replicated via a reduced score in NHS and showed greater diabetes reduction with an olive oil-rich Mediterranean diet among those with unfavorable baseline profiles in PREDIMED [61]. By contrast, SFA → MUFA substitutions showed weaker associations, plausibly reflecting local food matrices in which MUFA co-travels with SFA (mixed animal-source dishes) rather than plant oils. PRAL was substantially higher in men, consistent with greater animal-protein/phosphate exposure and lower plant-mineral density [62]. As expected in a young, relatively healthy sample and given the cross-sectional design, we observed no material substitution effects on SBP, FG or WHtR. Taken together, these data support fat-quality improvement (SFA → PUFA) as a feasible, modifiable lever; however, estimates should be interpreted as programmatic surveillance signals, not causal clinical effects.

Beyond the biological interpretation, these results carry direct implications for surveillance-linked prevention and public health strategy. The broader exposure environment appears to compound risk in this age group. To our knowledge, this is the first description of physical-activity patterns in Albanian young adults [14], addressing a national data gap and establishing a platform for ongoing monitoring. Within this cohort, prolonged sitting was common and intakes of several vitamins (D, E, folate) and minerals (potassium, calcium, iodine, magnesium) were below established benchmarks, while sodium exceeded WHO targets. This constellation is consistent with pathways linking visceral adiposity, insulin resistance and hepatic steatosis and highlights modifiability: routine waist measurement and BP assessment, coupled with clear counseling on fat quality (SFA → PUFA), dietary fiber, lower sodium and free sugars, represent pragmatic levers for surveillance-linked prevention in university-enrolled young adults. From a nutritional-epidemiology standpoint, additional 24 h recalls and calibration to objective biomarkers (e.g., urinary Na/K, fatty-acid biomarkers) would strengthen measurement validity; nonetheless, the current pattern is epidemiologically concerning as a consistent signal and warrants further investigation through prospective follow-up.

Limitations merit emphasis. The design is cross-sectional and the sample represents a sentinel university cohort rather than a population-based one; hence, causal inference is not intended. The cohort composition—predominantly women (65.6%)—reflects the broader feminization of higher education in Albania (64%) [63]. However, this pattern diverges from national enrollment profiles.

According to INSTAT, only 2.2% of all Albanian university students in 2024 were enrolled in agriculture, forestry, fisheries and veterinary sciences—fields that remain traditionally male-dominated, with women representing 43.6% of students [63]. This contextual contrast underscores that the *NutriSYN* cohort, while not population-representative by design, captures an academically distinct and strategically relevant segment situated at the intersection of agricultural education and public health. The findings are therefore not intended to generalize to all Albanian youth but to provide early insights into an underrepresented academic group with growing importance for the country’s agri-food and nutrition transition.

Furthermore, self-reported diet and predictive equations introduce measurement error that likely attenuates associations toward the null. Also, IPAQ-SF is still with no formal Albanian validation completed to date and we hope our results encourage validation. Nonetheless, the standardized protocol, high data completeness and a priori modeling support internal consistency and the sentinel design prioritizes reproducibility over time.

Looking forward, the *NutriSYN* platform is primed for longitudinal follow-up with biomarker calibration (e.g., urinary Na/K), device-based activity and expanded sampling to refine inference and evaluate the real-world impact of upstream measures. From a public health perspective, even modest, consistently observed shifts in BP distributions or nutrient-pattern indicators justify low-regret preventive actions—notably salt reduction, improvements in fat and fiber quality and environments that support movement—implemented within a surveillance framework capable of tracking uptake and equity. Nevertheless, our study demonstrates the feasibility of WHO-aligned sentinel surveillance in a previously under-monitored group and provides a transparent baseline for monitoring diet–cardiometabolic indicators in Albanian young adults. The subphenotype we describe—central adiposity with vascular and glycemic drift—offers a practical target for early detection and programmatic response, to be tested and refined through prospective replication rather than inferred causally from a single cross-section.

## 5. Conclusions

This study demonstrates the feasibility of WHO-aligned, cross-sectional surveillance in Albania, establishing (to our knowledge) the first sentinel baseline in young adults that integrates interviewer 24 h dietary recalls, a physical-activity instrument and a benchmark-referenced panel of CM and macro-/micronutrient indicators. Within an ostensibly BMI-normal university cohort, we identified a waist-centric CM subphenotype—*central adiposity co-occurring with upward BP shifts and impaired fasting glycemia*—that BMI alone does not detect. In prespecified energy-partition models, reallocating SFA → PUFA was associated with more favorable nutrient signatures (lower PRAL and a more favorable MASLD-oriented score), indicating a pragmatic, modifiable target within surveillance frameworks. These observations are presented as baseline surveillance signals—not causal effects and not population-level estimates. Embedding the metrics in routine information systems can support accountability and align with WHO “Best Buys” (salt reduction, improved fat quality, lower free sugars) alongside youth-supportive activity environments. The NutriSYN platform is primed for longitudinal follow-up; biomarker calibration (e.g., urinary Na/K), device-based activity and periodic replication will be essential to confirm reproducibility, refine inference and inform scalable prevention and policy over time.

## Figures and Tables

**Table 1:** Participant characteristics by sex.

Variable (Unit)		Women (N = 172)	Men (N = 90)	Total
Age (years)		21 (21–22)	21 (21–22)	21 (21–22)
Height (cm)		163.5 (159.0–168.0)	177.0 (173.0–184.0)	167.0 (161.0–174.0)
Weight (kg)		59.03 (58–61.50)	74.50 (68.50–77.50)	64.43 (61.70–66.35)
Waist circumference (cm)		75 (73.30–77)	88.18 (84–90.75)	79.25 (77.75–81)
Hip circumference (cm)		99.82 (98.50–101)	102.75 (100–106.50)	100.38 (99.50–101.80)
WHtR		0.46 (0.43–0.50)	0.51 (0.46–0.53)	0.47 (0.43–0.51)
BMI (kg/m^2^) *		24.08 (23.03–25.12)	22.98 (22.42–23.55)	23.36 (22.84–23.87)
SBP (mmHg)		109.0 (100.0–110.0)	120.0 (110.0–128.8)	110.0 (105.0–120.0)
DBP (mmHg)		70.0 (70.0–80.0)	80.0 (75.0–85.0)	75.0 (70.0–80.0)
MAP (mmHg)		83.33 (83.33–86.67)	90.83 (88.33–95)	86.67 (86.67–88.33)
Blood glucose (mg/dL)		95.2 (88.7–103.0)	102.0 (95.3–107.4)	97.7 (90.7–105.0)
Total PA time (min/week)		1676 (1386–1979.50)	2821.50 (1872.50–3465)	1923.75 (1626–2238)
Sitting time (min/day)		480 (480–540)	450 (420–480)	450 (450–480)
PA energy expenditure (kcal/day)		1742.93 (1358–1986.50)	3542.25 (2191.04–4439)	1996.48 (1740–2214)
Essential amino acids (g/1000 kcal)		19.73 (18.41–21.37)	22.93 (21.17–25.25)	21.08 (19.76–21.94)
Alcohol intake ** (drinks/day)		0.03 (max 1.4), 29.7% any	0.17 (max 2.6), 35.6% any	0.08 (max 2.6), 31.7% any
PRAL (mEq/day)		14.50 (10.46–20.29)	32.58 (24.98–40.04)	20.46 (15.37–25.56)
WCRF score (points)		5.25 (5.25–6.12)	4.81 (4.38–5.25)	5.25 (5.25–6.12)
Life’s Essential 8 (0–100)		54.17 (50–58.33)	50 (50–58.33)	50 (50–58.33)
cCMRS (z-score)		−0.27 (−0.37 to −0.16)	0.38 (0.24–0.49)	−0.05 (−0.16–0.04)
MASLD nutrient score (points)		2 (2–3)	2 (2–3)	2 (2–3)
WHtR ≥ 0.5 (*n*/%)		42 (24.4%)	46 (51.1%)	88 (33.6%)
IDF (≥94 cm men, ≥80 cm women)		54 (31.4%)	32 (35.6%)	86 (32.8%)
Nicotine use	No	160 (69.3%)	71 (30.7%)	231 (100.0%)
	Yes	12 (38.7%)	19 (61.3%)	31 (100.0%)
Habitual activity level	Low	66 (62.3%)	40 (37.7%)	106 (100.0%)
	Moderate	80 (76.9%)	24 (23.1%)	104 (100.0%)
	High	26 (50.0%)	26 (50.0%)	52 (100.0%)

*Notes*: SBP/DBP, systolic/diastolic BP; MAP, mean arterial pressure (mm Hg); WHtR, waist-to-height ratio; IDF, International Diabetes Federation cut-offs; BMI, body-mass index (kg/m^2^); PA, physical activity; PRAL, potential renal acid load (mEq/d); WCRF/AICR score (0–7, higher better); MASLD nutrient score (MASLD proxy, higher worse); LE8, modified Life’s Essential 8 (diet, PA, nicotine, BMI, BP, glucose; sleep excluded), scored 0–100 for available components, non-canonical LE8; cCMRS, composite cardiometabolic risk score. * BMI reported as mean (95% CI); others as median (IQR). ** Values shown as mean (max), % with any intake.

**Table 2:** Isocaloric nutrient substitutions and cardiometabolic markers.

Food Sources Substituted (Orientation) † 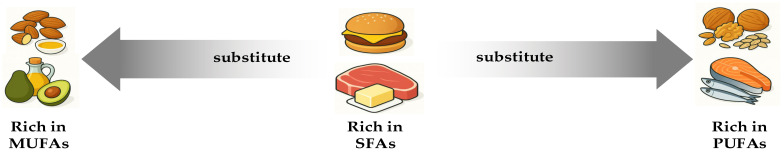					
Domain	Outcome	Substitution (from → to)	Effect per +5%E (95% CI)	*p* Value	q (BH)
***Metabolic*** ***signatures***	PRAL (%)	SFA (%E) → PUFA (%E)	−32.8% (−56.6, −9.1)	0.007	0.054
	PRAL (%)	SFA (%E) → MUFA (%E)	−20.9% (−42.7, +0.8)	0.060	0.238
	MASLD	SFA (%E) → PUFA (%E)	−28.2% (−39.0, −17.4)	<0.001	<0.001
	MASLD	SFA (%E) → MUFA (%E)	−12.4% (−22.3, −2.6)	0.013	0.070
***Clinical*** ***signatures***	SBP (mmHg)	SFA (%E) → PUFA (%E)	−1.00 mmHg (−2.88, +0.90)	0.300	0.791
	SBP (mmHg)	SFA (%E) → MUFA (%E)	−0.57 mmHg (−1.88, +0.75)	0.396	0.791
	Glucose (mg/dL)	SFA (%E) → PUFA (%E)	+0.40 mg/dL (−1.58, +2.41)	0.696	0.827
	Glucose (mg/dL)	SFA (%E) → MUFA (%E)	+0.23 mg/dL (−1.46, +1.96)	0.788	0.841
	WHtR	SFA (%E) → MUFA (%E)	−0.6% (−2.5, +1.4)	0.583	0.827
	WHtR	SFA (%E) → PUFA (%E)	−0.7% (−4.2, +3.0)	0.711	0.827
***Composite*** ***signatures***	Life’s Essential 8 (SD)	SFA (%E) → PUFA (%E)	+0.11 SD (−0.13, +0.36)	0.386	0.791
	Life’s Essential 8 (SD)	SFA (%E) → MUFA (%E)	+0.06 SD (−0.12, +0.25)	0.509	0.827
	WCRF (SD)	SFA (%E) → PUFA (%E)	+0.09 SD (−0.08, +0.26)	0.303	0.791
	WCRF (SD)	SFA (%E) → MUFA (%E)	+0.02 SD (−0.10, +0.14)	0.724	0.827
	cCMRS (SD)	SFA (%E) → MUFA (%E)	−0.03 SD (−0.17, +0.11)	0.667	0.827
	cCMRS (SD)	SFA (%E) → PUFA (%E)	−0.01 SD (−0.20, +0.19)	0.929	0.929

*Note:* Estimates reflect isocaloric +5%E reallocations (energy-partition models, carbohydrate omitted), adjusted for age, sex, smoking and physical activity with HC3 SEs. Associations are exploratory and not intended as clinical effect estimates. Lower PRAL/MASLD and higher LE8/WCRF indicate more favorable profiles. Multiple testing controlled by Benjamini–Hochberg FDR (q shown). Abbreviations: SFA, PUFA, MUFA, PRAL, MASLD nutrient score, LE8, cCMRS, SBP, WHtR. ^†^ Illustration intended for interpretive clarity.

## Data Availability

Data underlying this study are available from the senior authors (E.L. and V.G.) upon reasonable request. Access will be governed by a data use agreement and provided in accordance with conditions approved by the senior authors.

## Supplementary Materials

The following supporting information can be downloaded: https://www.mdpi.com/article/10.3390/nu17213395/s1.

## Abbreviations

The following abbreviations are used in this manuscript:

**Abbreviation**	**Definition**
*ALA/C18:3*	α-Linolenic Acid (per 1000 kcal)
*BMI*	Body Mass Index (kg/m^2^)
*cCMRS*	Composite Cardiometabolic Risk Score (standardized)
*C16:0*	Palmitic Acid (per 1000 kcal)
*C18:1*	Oleic Acid (per 1000 kcal)
*C18:2*	Linoleic Acid (per 1000 kcal)
*DBP*	Diastolic Blood Pressure (mmHg)
*EAA*	Essential Amino Acids (per 1000 kcal)
*EI/TEE*	Energy Intake/Total Energy Expenditure ratio
*EPA/1000 kcal*	Eicosapentaenoic Acid (per 1000 kcal)
*FDR (q)*	False Discovery Rate (Benjamini–Hochberg)
*HBSC*	Health Behavior in School-aged Children survey (WHO/Europe)
*IDF*	International Diabetes Federation
*IQR*	Interquartile Range
*K/1000 kcal*	Potassium (per 1000 kcal)
*LE8*	Life’s Essential 8 cardiovascular health score (AHA, 0–100 scale)
*MET*	Metabolic Equivalent of Task
*MUFA*	Monounsaturated Fatty Acids
*NCDs*	Non-Communicable Diseases
*PRAL*	Potential Renal Acid Load (mEq/day)
*PUFA*	Polyunsaturated Fatty Acids
*SBP*	Systolic Blood Pressure (mmHg)
*SFA*	Saturated Fatty Acids
*WCRF score*	World Cancer Research Fund index score
*WHtR*	Waist-to-Height Ratio
