# Supplementary material for: Cardiometabolic Phenotypes and Dietary Patterns in Albanian University-Enrolled Young Adults: Cross-Sectional Findings from the Nutrition Synergies WHO-Aligned Sentinel Platform
Source: Nutrients. 2025 Oct 29;17(21):3395. doi: 10.3390/nu17213395 (PMC12608412; doi:10.3390/nu17213395)
Supplement: Supplementary file 1 [file nutrients-17-03395-s001.zip › nutrients-3956074-supplementary.pdf]

nutrients

MDPI

Supplementary material

Cardiometabolic Phenotypes and Dietary Patterns in Albanian University-Enrolled Young Adults: Cross-Sectional Findings from the *Nutrition Synergies* WHO-Aligned Sentinel Platform

Vilma Gurazi[1] †*, Sanije Zejnelhoxha[1] †, Megisa Sulenji[1] ‡, Lajza Koxha[1] ‡, Herga Protoduari[1] ‡, Kestjana Arapi[1] ‡, Elma Rexha[1] ‡, Flavia Gjata[1] ‡, Orgesa Spahiu[1] ‡ and Erand Llanaj[2,3,4,5,6] *

Contents

STROBE / STROBE-nut Compliance Matrix 2
Supplementary methods: Intake–expenditure plausibility 4
Energy-Partition Model (Isocaloric Substitution Framework) 5
Sensitivity analyses 10
NutriSYN framework 12
S1. Overview and Rationale (expanded) 14
Principles. 15
S2. Data Preparation and Unit Harmonization 15
S2.1. Data Infrastructure and Versioning 15
S2.2. Anthropometric and Clinical Data 15
Measurement Procedures 15
Blood Pressure 16
Fasting Glycemia 16
S2.3. Dietary Data 16
Collection and Entry 16
Portion Size Estimation 17
Quality Control 17
S2.4. Harmonization of Nutrient Units 17
S3. Dietary Renal Acid Load (PRAL) 17
S3.1. Conceptual Basis 17
S3.2. Computational Formula 18
S3.3. Data Inputs and Harmonization 18
S3.4. Analytical Treatment 18
S3.5. Interpretation and Epidemiologic Use 19
S3.6. Validation and Sensitivity Checks 19
S3.7. Integration with Other Indices 19
S3.8. Strengths and Limitations of PRAL Application 19
Summary: 20
S4. MASLD-Oriented Nutrient Score 20
S4.1. Conceptual Rationale 20
S4.2. Construct and Component Selection 20
S5. WCRF/AICR Adherence Score 21
S5.1. Conceptual Foundation 21
S5.2. Index Structure and Scoring Logic 22
S6. Modified Life's Essential 8 (LE8) Score 22
S6.1. Conceptual Framework 22
S6.2. Scoring Approach and Computational Method 23
S7. Composite Cardiometabolic Risk Score (ccmrs) 23
S7.1. Rationale and Conceptual Framework 23
S7.2. Components and Calculation 24
S8. Data Quality Control and Reliability Framework 24
S8.1. Rationale and Governance 24
S8.2. Quality-Assurance Architecture 24

S9. Statistical Modeling Framework........25
S9.1. Analytical Philosophy........25
S9.2. Model Type: Energy-Partition Substitution Framework........25
S9.3. Pre-Specified Substitution Contrasts........26
S9.4. Outcome Families and Multiple-Comparison Control........26
S10. Longitudinal Expansion and Future Calibration Plan........26
S10.1. Vision and Strategic Rationale........26
S10.2. Follow-up Architecture........27
Design........27
Retention and Tracking........27
S10.3. Planned Calibration Enhancements........27
S11. Limitations, Replicability, and Methodological Safeguards........28
S11.1. Overview........28
S11.2. Study Design and Generalizability........28
S11.3. Sample Size and Power........28
S11.4. Cross-Sectional Constraints and Temporality........29
S11.5. Dietary Assessment and Recall Bias........29
S11.6. Energy Partition and Substitution Modeling........29
S11.7. Measurement Error in Clinical Markers........30
S11.8. Residual Confounding and Covariate Adjustment........30
S11.9. Replicability and Analytical Transparency........30
S11.10. Interpretive Boundaries and Surveillance Role........31
S11.11. Strengths as Inverse of Limitations........31
S11.12. Summary........31
S12. Integration with Policy Translation and Health-System Uptake........31
S12.1. Translational Rationale........31
S12.2. Linkage to WHO and EU Frameworks........32
S13. Communication, Dissemination, and Public Engagement........32
S13.1. Translational Philosophy........32
S13.2. Communication Architecture........32
S13.3. Core Communication Products........33
1. Policy Briefs and Executive Summaries........33
2. Annual Sentinel Report........33
3. Media Engagement Toolkit........33
4. Public-Facing Infographics........33
S14. Integration of the University of Medicine and Other National Institutions: Expanding Expertise, Scope, and Collaboration in Albania........33
S14.1. Strategic Imperative for Institutional Integration........34

STROBE / STROBE-nut Compliance Matrix

Domain	Item (STROBE / STROBE-nut requirement)	Where addressed in our work	Notes / highlights

Title & Abstract	Indicate exposure(s), outcome(s), design, population; avoid unexplained abbreviations	Title & Abstract, revised ms. (Article header + Abstract)	Title specifies cardiometabolic phenotypes, diet/activity, young adults, and cross-sectional sentinel baseline; abstract reflects the same.
Background / Rationale	Scientific context and justification	Intro (rationale paragraph opening of revised ms.)	Frames nutrition transition, surveillance gap, and sentinel approach.
Objectives	Prespecified aims / hypotheses	Intro (closing paragraph), Methods overview	Establishes surveillance-oriented, hypothesis-generating aim and substitution focus (SFA→PUFA).
Study Design	Early in paper; key design features	Methods: "Study design and setting" (cross-sectional, WHO-aligned sentinel)	"Wave-1 (baseline)" clarified as descriptive/surveillance.
Setting	Locations, dates, setting details	Methods: Setting & recruitment; flow diagram reference	On-campus clinics; pragmatic sampling; STROBE flow noted.
Participants	Eligibility, recruitment, sample derivation	Methods: "Participants and recruitment" + flow diagram reference	Prespecified eligibility; screening logs; no post-hoc outcome-based exclusions.
Ethics	Approvals, consent	Methods: Ethics paragraph (AUBT EC #3400)	Written consent; Declaration of Helsinki.
Variables (clinical)	Clearly define outcomes, exposures, confounders	Methods: Anthropometry, BP, FG definitions & cut-points	ESC/ESH BP, IDF waist cut-offs; ADA glycemia bands; cCMRS components defined.
**Dietary Assessment (STROBE-nut)**	Method, instrument, interview mode, recall days, quality control	Methods: Dietary assessment & lifestyle	Two interviewer 24-h recalls, Multiple-Pass Method, SOPs, real-time plausibility checks.
**Portion-size Estimation (STROBE-nut)**	Portion tools & local adaptation	Original + revised detail (atlas + anchors)	Culturally adapted pictorial atlas and local gram-weight tables.
**Food-Composition Data (STROBE-nut)**	Database(s), version, cross-checks	Methods: nutrient derivation (NutriSurvey + McCance & Widdowson cross-check)	Custom Albanian FCD, harmonization with McCance & Widdowson.
**Energy Adjustment & Units (STROBE-nut)**	How nutrients were scaled/standardized	Methods: energy adjustment conventions; %E, g/1000 kcal; density metrics	Aligns with nutritional-epi standards.
**Diet-Quality / Pattern Indices (STROBE-nut)**	Precisely define indices; algorithms; orientation	Methods + Supplement (PRAL; MASLD nutrient score; WCRF; LE8; cCMRS)	Components, directionality, computation and rationale detailed; primary FDR family stated.
**Usual Intake / Misreporting (STROBE-nut)**	Misreporting assessment; handling	Methods: Goldberg EI:TEE, no trimming (descriptive only). Supplement S-Methods (Goldberg figure)	Explains bias trade-off in small strata; energy-density metrics mitigate.
Data Sources / Measurement	Devices, calibration, duplicate measures, training	Methods: SOPs; duplicate anthropometry & BP; trained assessors	QC/QA described; supports reliability.
Bias	Efforts to address measurement/confounding bias	Methods: prespecified covariates; misclassification discussion; FDR	
Study Size	How size was arrived at; analytic yield	Results: flow and denominators (final n=262) + Methods flow ref.	Screens → eligible → analytic; minimal missingness noted.

Quantitative Variables	Handling/transformations	Methods: log-transform for PRAL/MASLD/WHtR; z-scores; units	
Statistical Methods	All analyses; multiplicity; diagnostics	Methods: energy-partition models; +5%E substitutions; BH-FDR; robust SEs; VIF; residual/influence checks	
Participants (Results)	Flow of participants; characteristics	Results table/fig references in ms. (sex-stratified summaries)	Sex-specific medians (IQR); BMI normal on average.
Descriptive Data (Results)	Key exposure/outcome distributions	Results narrative + figures (indices, nutrients, BP/glycemia/waist)	
Outcome Data	Prevalence/levels by strata	Results (BP, FG categories; waist measures)	Clear clinical cut-points used for context only (non-diagnostic).
Main Results	Unadjusted/adjusted & precision; multiplicity	Results + Table of substitutions	
Other Analyses	Sensitivity / visualization choices	Supplement: S-Methods; Figures S1–S2; rationale	
Limitations	Key biases/limits and direction	Discussion / Methods notes	
Interpretation	Cautious, consistent with results & limits	Discussion (non-causal, surveillance tone)	"Signals" language; feasibility emphasized.
Generalizability	External validity; sentinel role	Discussion (sentinel utility; replication plan)	Positioned as baseline for trend monitoring, not population estimates.
Funding / Roles	Funding and author roles (if present)	End of article	Included funder statements
**STROBE-nut: Interviewer / Training**	Who collected diet data; training; QA	Methods (trained assessors, SOPs)	Interviewer standardization described.
**STROBE-nut: Physical Activity**	Instrument validity/translation	Methods (IPAQ-SF forward/back translation)	Surveillance-appropriate; limitation acknowledged elsewhere.
**STROBE-nut: Index Justification**	Why PRAL, MASLD, WCRF, LE8, cCMRS	Methods + Supplement sections with rationale and constructs	Mechanistic domains; hepatic/renal coupling; composite CM signal.
**STROBE-nut: Misreporting**	Approach to detect/handle EI misreporting	Methods + Supplement (Goldberg categories; no exclusions)	Used descriptively to preserve comparability in small strata.
**STROBE-nut: Reproducibility**	Versioning, algorithms, transparency	Supplement contents and algorithm boxes	

Referecne: Lachat C, Hawwash D, Ocké MC, Berg C, Forsum E, Hörnell A, Larsson C, Sonestedt E, Wirfält E, Åkesson A, Kolsteren P, Byrnes G, De Keyzer W, Van Camp J, Cade JE, Slimani N, Cevallos M, Egger M, Huybrechts I. Strengthening the Reporting of Observational Studies in Epidemiology-Nutritional Epidemiology (STROBE-nut): An Extension of the STROBE Statement. PLoS Med. 2016 Jun 7;13(6):e1002036. doi: 10.1371/journal.pmed.1002036. PMID: 27270749; PMCID: PMC4896435.

Supplementary methods: Intake–expenditure plausibility

Self-reported total energy intake (TEI) aligned with estimated total energy expenditure (TEE): the TEI–TEE relation centered near the identity with modest dispersion (Figure S1). Sex-specific OLS fits had shallow, overlapping slopes, indicating no material sex difference in TEI–TEE scaling at this sample size. In line with known reporting patterns, TEI medians were higher in women (2269 vs 1891 kcal/day), whereas TEE medians were higher in men; distributions overlapped.

Goldberg EI: TEE categories were used descriptively (no trimming) to avoid selection bias; given this context, the apparent TEI contrast is most plausibly attributable to reporting bias rather than true physiologic difference. Energy adjustment and density-based metrics were used in primary analyses.

Figure S1. Energy-reporting concordance (intake vs. Expenditure), by sex.

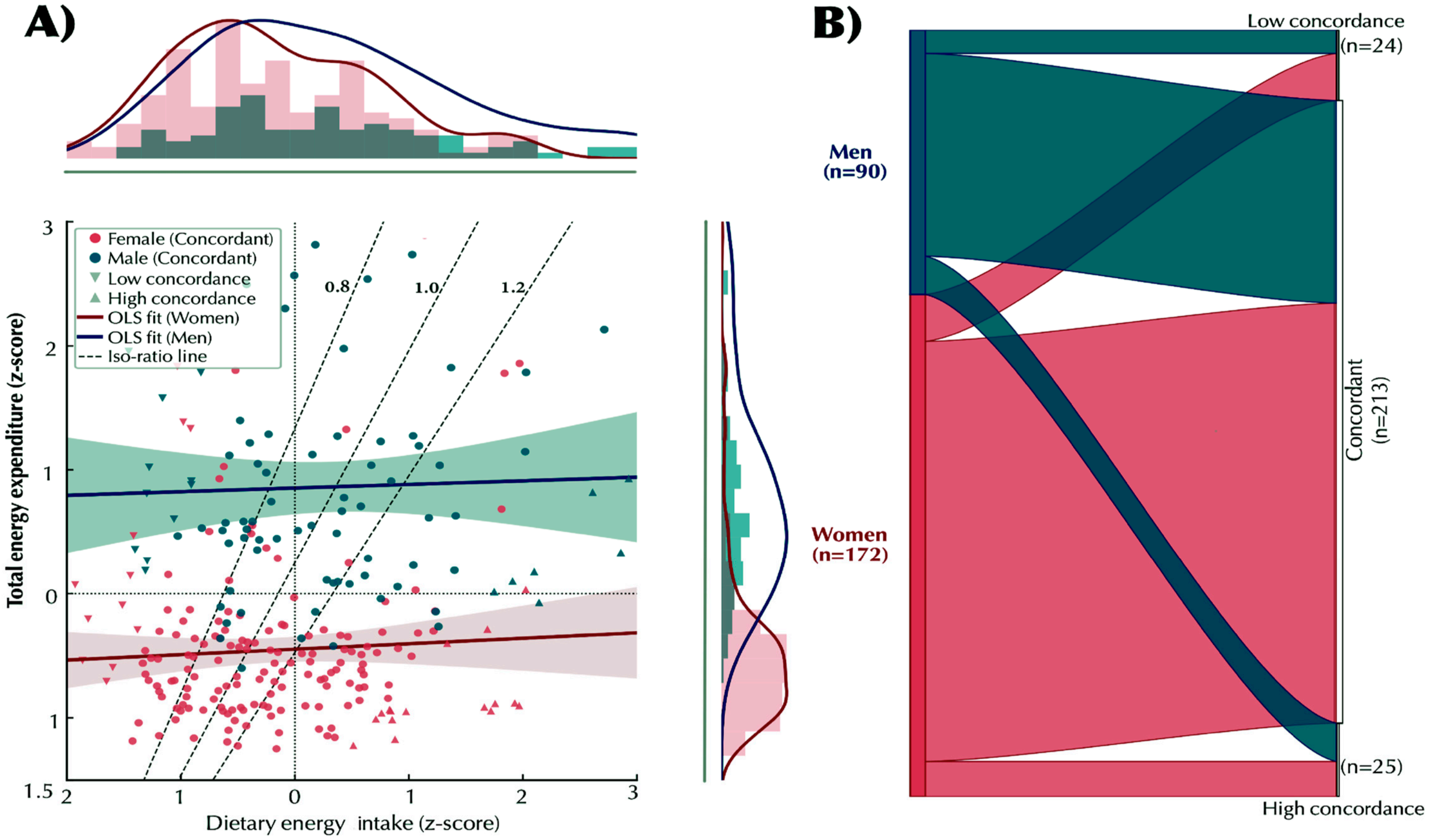

Note: Panel A: Z-scored total energy intake (TEI, x-axis) vs expenditure (TEE, y-axis) sex-specific OLS fits ±95% CI; marginal histograms with density overlays. Panel B: Sankey of sex and Goldberg EI/TEE categories: low (<0.8), concordant (0.8–1.2), high (>1.2); node/flow size ∝ count. TEI, TEE as defined in Methods; Goldberg categories shown for context only (no data excluded).

Energy-Partition Model (Isocaloric Substitution Framework)

This model (Scheme S1)quantifies how reallocating small proportions of daily energy among macronutrients relates to selected metabolic outcomes, while keeping total energy intake constant. All energy-yielding nutrients—saturated (SFA), monounsaturated (MUFA), and polyunsaturated fatty acids (PUFA), protein, alcohol, and carbohydrate—enter the regression simultaneously.

Carbohydrate is omitted from the model, serving as the reference category against which substitutions are interpreted. Each β-coefficient thus represents the expected difference in the outcome (e.g., systolic blood pressure, fasting glucose, PRAL, MASLD nutrient score, waist-to-height ratio, LE8, WCRF score, or composite cardiometabolic risk score) associated with a 1% energy increase from that nutrient while proportionally decreasing carbohydrate energy. To estimate meaningful contrasts, we modeled +5% energy reallocations from SFA to PUFA and from SFA to MUFA—conceptually equivalent to replacing butter or fatty meats with vegetable oils, nuts, or olives under stable total calorie intake. Analyses adjusted a priori for age, sex, smoking status, physical activity, and total energy intake, reflecting common confounders of diet–disease associations. The resulting β-coefficients (and 95% CIs)

describe population-level associations, not causal effects, and are interpreted as hypothesis-generating signals for future longitudinal confirmation.

Scheme S1. Isocaloric Substitution Framework

ENERGY-PARTITION MODEL (ISOCALORIC SUBSTITUTION)

All energy-yielding macronutrients included simultaneously:

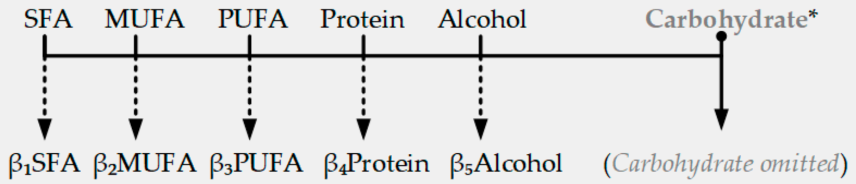

Regression form:

$$Outcome = \beta_0 + \beta_1(SFA) + \beta_2(MUFA) + \beta_3(PUFA) + \beta_4(Protein) + \beta_5(Alcohol) + \Sigma Covariates + \varepsilon$$

Substitution contrasts (Δ5%E):

- +5%E SFA → PUFA → $\beta_3 - \beta_1$
- +5%E SFA → MUFA → $\beta_2 - \beta_1$

Covariate set (*a priori*): age, sex, smoking, physical activity, energy intake

Outcomes *(selected):*
Systolic blood pressure, fasting glucose, PRAL, MASLD-score, WHtR, LE8, WCRF, cCMRS

Outputs: β-coefficients per 5%E substitution, 95% CI, FDR-adjusted p-value

**Carbohydrate omitted as reference in all isocaloric contrasts.*

Correlation heat maps (Figure S2A) showed positive co-variation among whtr, BP, fasting glycemia and ccmrs. WCRF and LE8 inversely correlated with ccmrs, PRAL and the MASLD-oriented score, whereas plant-forward nutrient density (e.g., potassium, magnesium, folate, vitamin C) grouped with more favorable profiles. Sodium correlated positively with BP-proximal markers. These patterns reinforce a clustered, waist-centric CM profile in early adulthood, coherent with the distributions above.

Figure S2. Pairwise correlations among diet-quality indices, nutrient densities, physical activity and cardiometabolic markers.

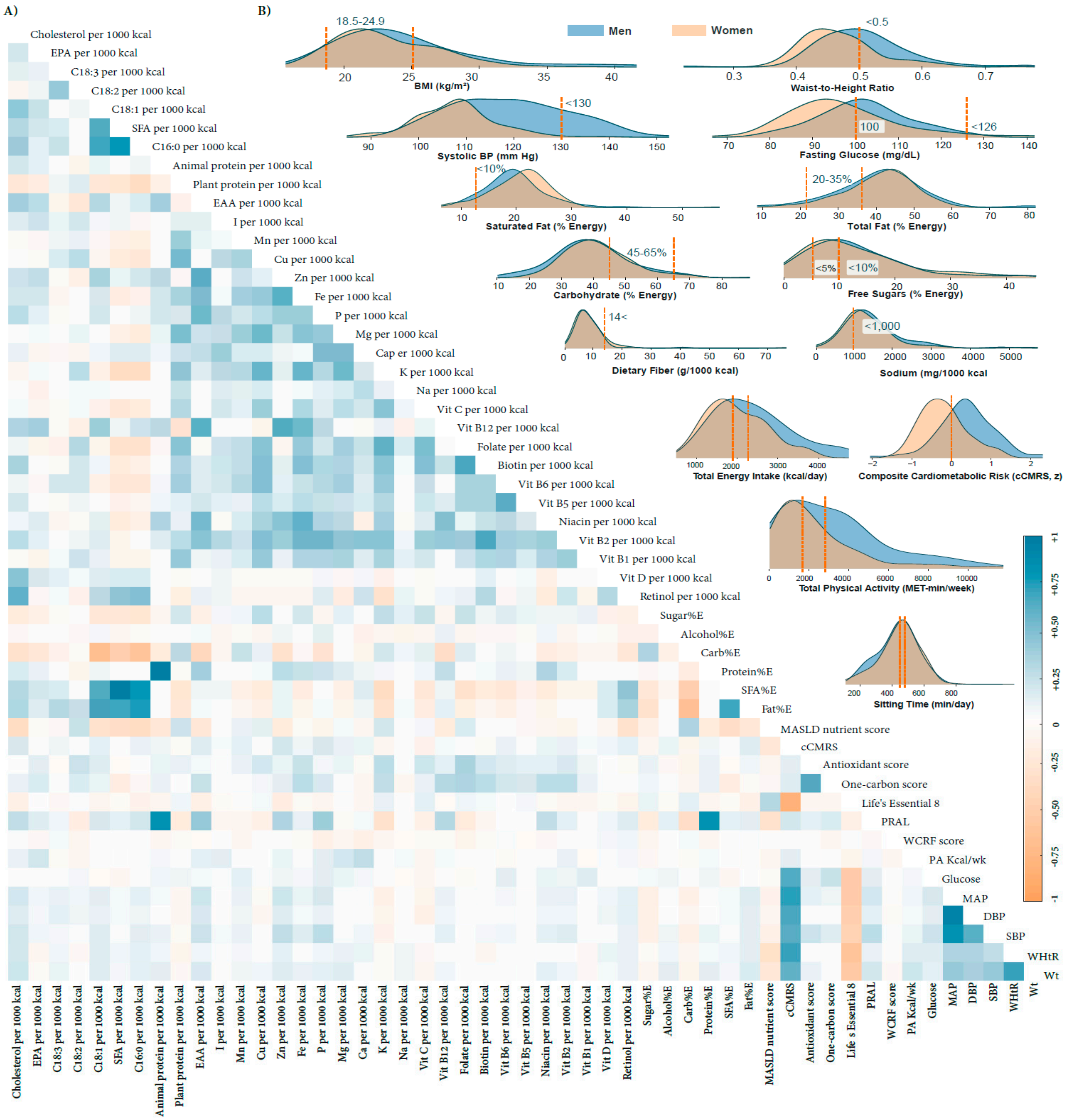

Note: Panel A: Correlation matrix of nutrients, anthropometry, cardiometabolic parameters and composite scores; shading reflects Pearson coefficients (blue = positive orange = negative). Panel B: Sex-stratified kernel density plots (standardized); dashed lines indicate clinical/dietary thresholds (men = blue, women = orange). Abbreviations: %E, percent energy; PRAL, potential renal acid load (meq/day); MASLD nutrient score (MASLD proxy); WCRF/AICR, dietary adherence; LE8, Life's Essential 8; ccmrs, composite cardiometabolic risk score.

Figure S3. Vitamin intake distributions by sex.

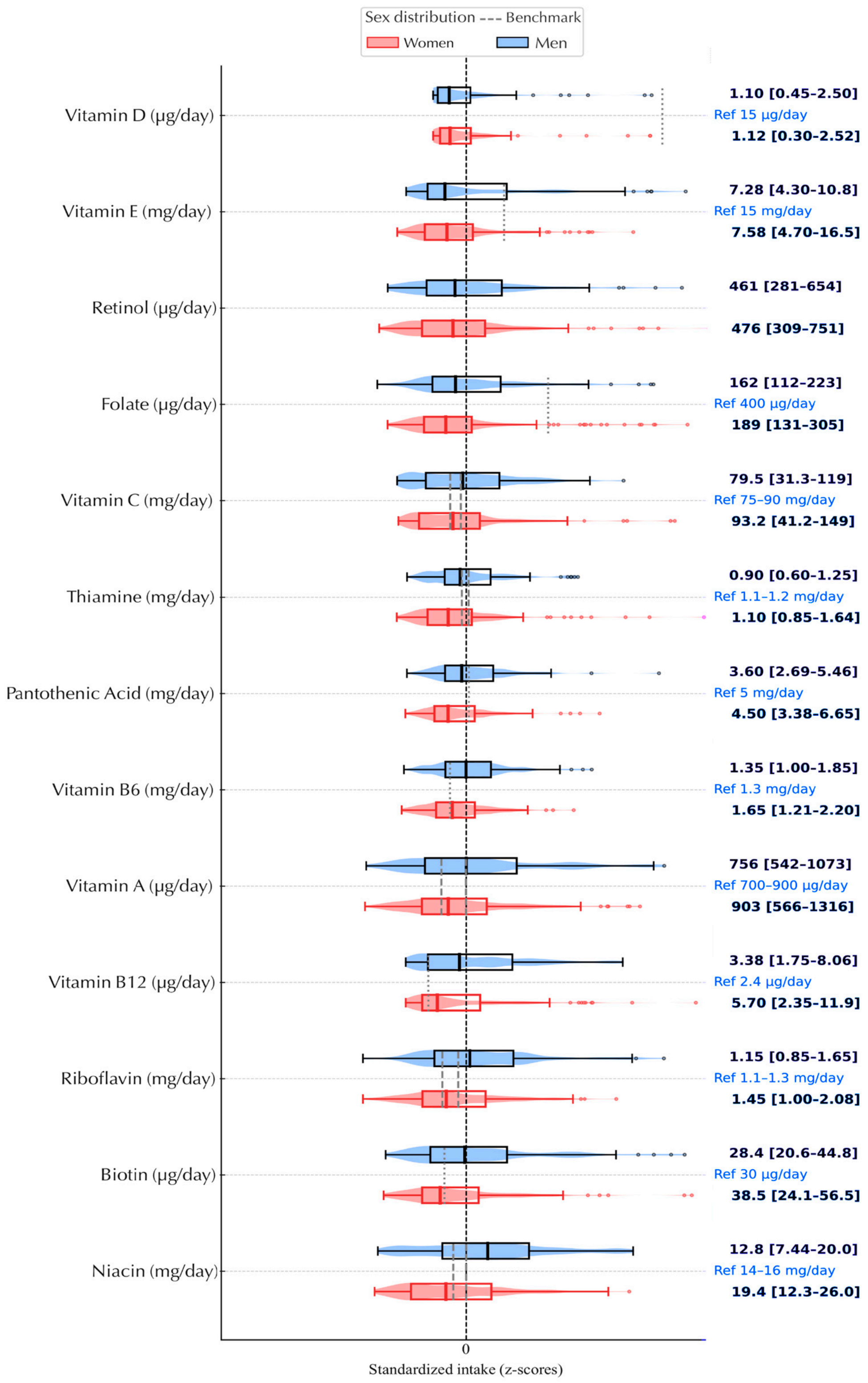

Note: Violin plots depict sex-specific distributions; interior boxes show medians (center line) and interquartile ranges (IQR). Reference lines indicate commonly cited recommended intakes (sex-specific where applicable). The x-axis shows standardized intake (z-scores) for plotting; interpretation relies on the raw-unit alongside each row (mg/day or µg/day, as labeled). Retinol is displayed separately from vitamin A.
Figure S4. Mineral intake distributions by sex.

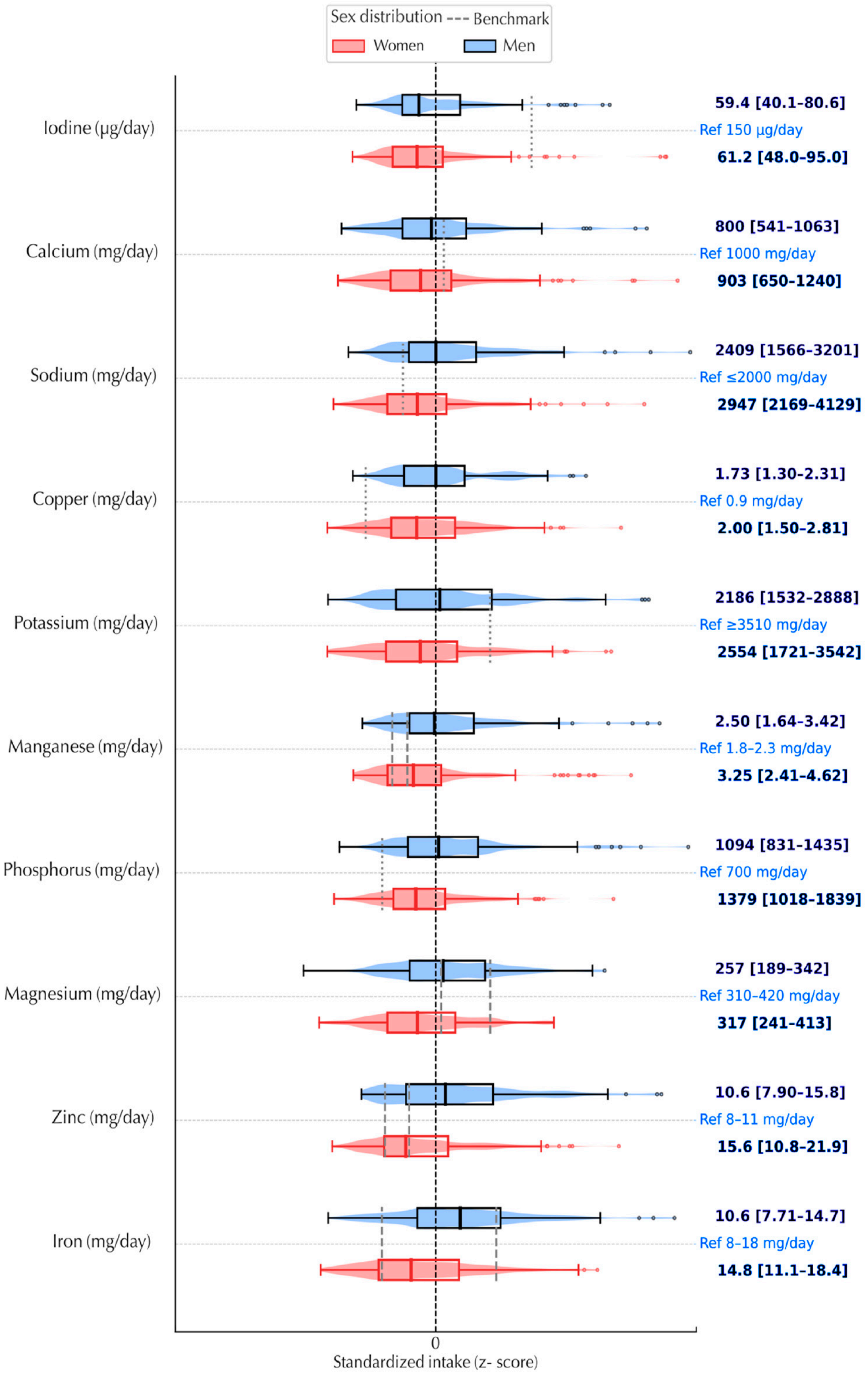

Note: Violin plots display sex-specific distributions. The x-axis shows standardized intake (z-scores) for plotting; interpretation relies on the raw-unit medians [IQR] alongside each row (mg/day or µg/day). Vertical reference lines denote commonly used recommended intakes (sex-specific where shown): iron 8/18 mg/day, zinc 8/11 mg/day, magnesium 310/420 mg/day, potassium 3510 mg/day, sodium 2000 mg/day, calcium 1000 mg/day and iodine 150 µg/day.

This appendix provides a full, step-by-step specification of all indices, transformations, scoring rules, quality-control (QC) steps, modeling frameworks, and sensitivity analyses used in the sentinel baseline. It is written so another group could

replicate the pipeline exactly, and so future nutrisyn waves can preserve time-series comparability.

Sensitivity analyses

Variants: (i) HC3 robust ses (baseline); (ii) cluster-robust ses (by center, if available); (iii) winsorization at 1st/99th percentiles; (iv) model without alcohol covariate. Effects are per +5%E reallocated, formatted in the same units as the main table.

Outcome	Swap	Variant	Effect (per +5%E)
SBP	SFA (%E) → PUFA (%E)	HC3	-1.00 mmhg (-2.88, +0.90)
SBP	SFA (%E) → PUFA (%E)	Winsorized (1–99%)	-0.31 mmhg (-3.05, +2.50)
SBP	SFA (%E) → PUFA (%E)	No alcohol (HC3)	-0.98 mmhg (-2.84, +0.91)
SBP	Carbohydrate (%E) → PUFA (%E)	HC3	-0.34 mmhg (-1.76, +1.09)
SBP	Carbohydrate (%E) → PUFA (%E)	Winsorized (1–99%)	-0.50 mmhg (-2.56, +1.59)
SBP	Carbohydrate (%E) → PUFA (%E)	No alcohol (HC3)	-0.30 mmhg (-1.70, +1.12)
PRAL	SFA (%E) → PUFA (%E)	HC3	-32.8% (-56.6, -9.1)
PRAL	SFA (%E) → PUFA (%E)	Winsorized (1–99%)	-48.0% (-67.5, -28.5)
PRAL	SFA (%E) → PUFA (%E)	No alcohol (HC3)	-33.8% (-58.3, -9.2)
PRAL	Carbohydrate (%E) → PUFA (%E)	HC3	-10.4% (-26.9, +6.0)
PRAL	Carbohydrate (%E) → PUFA (%E)	Winsorized (1–99%)	-20.9% (-35.4, -6.3)
PRAL	Carbohydrate (%E) → PUFA (%E)	No alcohol (HC3)	-12.3% (-29.6, +5.0)
Glucose	SFA (%E) → PUFA (%E)	HC3	+0.40 mg/dl (-1.58, +2.41)
Glucose	SFA (%E) → PUFA (%E)	Winsorized (1–99%)	+0.44 mg/dl (-2.45, +3.43)
Glucose	SFA (%E) → PUFA (%E)	No alcohol (HC3)	+0.38 mg/dl (-1.58, +2.39)
Glucose	Carbohydrate (%E) → PUFA (%E)	HC3	-0.00 mg/dl (-1.34, +1.35)
Glucose	Carbohydrate (%E) → PUFA (%E)	Winsorized (1–99%)	+0.38 mg/dl (-1.73, +2.54)
Glucose	Carbohydrate (%E) → PUFA (%E)	No alcohol (HC3)	-0.03 mg/dl (-1.34, +1.30)
MASLD	SFA (%E) → PUFA (%E)	HC3	-28.2% (+17.4, +39.0)
MASLD	SFA (%E) → PUFA (%E)	Winsorized (1–99%)	-33.0% (+20.1, +45.9)
MASLD	SFA (%E) → PUFA (%E)	No alcohol (HC3)	-28.1% (+17.3, +38.9)
MASLD	Carbohydrate (%E) → PUFA (%E)	HC3	-13.6% (+7.0, +20.3)
MASLD	Carbohydrate (%E) → PUFA (%E)	Winsorized (1–99%)	-17.7% (+7.3, +28.2)
MASLD	Carbohydrate (%E) → PUFA (%E)	No alcohol (HC3)	-13.5% (+6.8, +20.1)
Whtr	SFA (%E) → PUFA (%E)	HC3	-0.7% (-4.2, +3.0)

Whtr	SFA (%E) → PUFA (%E)	Winsorized (1–99%)	-0.5% (-4.1, +3.3)
Whtr	SFA (%E) → PUFA (%E)	No alcohol (HC3)	-0.7% (-4.3, +3.0)
Whtr	Carbohydrate (%E) → PUFA (%E)	HC3	+0.1% (-3.1, +3.4)
Whtr	Carbohydrate (%E) → PUFA (%E)	Winsorized (1–99%)	-0.2% (-3.3, +2.9)
Whtr	Carbohydrate (%E) → PUFA (%E)	No alcohol (HC3)	+0.1% (-3.1, +3.4)
LE8	SFA (%E) → PUFA (%E)	HC3	+0.11 SD (-0.13, +0.36)
LE8	SFA (%E) → PUFA (%E)	Winsorized (1–99%)	+0.09 SD (-0.17, +0.37)
LE8	SFA (%E) → PUFA (%E)	No alcohol (HC3)	+0.11 SD (-0.13, +0.36)
LE8	Carbohydrate (%E) → PUFA (%E)	HC3	+0.02 SD (-0.17, +0.21)
LE8	Carbohydrate (%E) → PUFA (%E)	Winsorized (1–99%)	+0.04 SD (-0.17, +0.26)
LE8	Carbohydrate (%E) → PUFA (%E)	No alcohol (HC3)	+0.01 SD (-0.17, +0.20)
WCRF	SFA (%E) → PUFA (%E)	HC3	+0.09 SD (-0.08, +0.26)
WCRF	SFA (%E) → PUFA (%E)	Winsorized (1–99%)	+0.04 SD (-0.20, +0.30)
WCRF	SFA (%E) → PUFA (%E)	No alcohol (HC3)	+0.09 SD (-0.08, +0.27)
WCRF	Carbohydrate (%E) → PUFA (%E)	HC3	+0.06 SD (-0.06, +0.19)
WCRF	Carbohydrate (%E) → PUFA (%E)	Winsorized (1–99%)	+0.10 SD (-0.09, +0.30)
WCRF	Carbohydrate (%E) → PUFA (%E)	No alcohol (HC3)	+0.07 SD (-0.06, +0.19)
Ccmrs	SFA (%E) → PUFA (%E)	HC3	-0.01 SD (-0.20, +0.19)
Ccmrs	SFA (%E) → PUFA (%E)	Winsorized (1–99%)	+0.03 SD (-0.21, +0.28)
Ccmrs	SFA (%E) → PUFA (%E)	No alcohol (HC3)	-0.01 SD (-0.20, +0.18)
Ccmrs	Carbohydrate (%E) → PUFA (%E)	HC3	+0.04 SD (-0.11, +0.18)
Ccmrs	Carbohydrate (%E) → PUFA (%E)	Winsorized (1–99%)	+0.04 SD (-0.14, +0.22)
Ccmrs	Carbohydrate (%E) → PUFA (%E)	No alcohol (HC3)	+0.04 SD (-0.11, +0.18)

Note: Cluster-robust column is omitted if a center/cluster variable was not detected. Winsorization applied to outcomes, exposures, and covariates symmetrically at 1st/99th percentiles. Directionality key: PRAL, MASLD and WHtR are modeled on the log scale and reported as back- transformed percent differences; negative values denote improvement (also stated in relevant table footnotes).

NutriSYN framework

The Figure S9 schematic illustrates the design logic of the **NutriSYN Sentinel Study**, which integrates nutritional, physiological, and contextual information to detect early cardiometabolic risk signatures among young adults in Albania. The framework is structured to translate dietary exposure data into interpretable cardiometabolic signals through a sequence of interlinked analytic layers.

1. Context and Confounding

Foundational sociodemographic and environmental determinants (age, sex, socioeconomic status, sleep, stress, seasonality, and food environment) provide the contextual substrate. These variables serve as covariates, recognizing their influence on both dietary behaviors and metabolic outcomes.

2. Data and Quality Control

Quantified dietary intake is obtained via two non-consecutive, interviewer-administered 24-hour recalls using the multiple-pass method (MPM). Physical activity and sedentary behavior are assessed by the *International Physical Activity Questionnaire–Short Form (IPAQ-SF)*. Basal metabolic rate (BMR) is calculated, allowing estimation of total energy expenditure (TEE) and identification of implausible energy reporters. The plausibility and misreport risk modules act as analytical filters ensuring data integrity before model estimation.

3. Dietary Pattern Constructs and Models

Energy-yielding macronutrients (SFA, MUFA, PUFA, protein, carbohydrate, alcohol) enter **isocaloric energy-partition models** simultaneously, with carbohydrate omitted as the reference nutrient. Substitution contrasts (+5%E SFA→PUFA; +5%E SFA→MUFA) estimate theoretical energy reallocations under constant total energy intake. Derived indices capture complementary dimensions of diet quality and metabolic load:

- **PRAL (Potential Renal Acid Load)** – reflects acid–base balance;
- **MASLD-oriented nutrient score** – captures lipotoxic and glucotoxic dietary signatures;
- **WCRF/AICR adherence score** – summarizes alignment with cancer-prevention guidelines;
- **Modified LE8 (Life's Essential 8)** – evaluates cardiovascular health based on available data.

4. Phenotypes and Risk Integration

Standardized phenotyping links dietary exposures to metabolic intermediates and early risk markers. Core outcomes include central adiposity (waist circumference, WHtR), blood pressure (BP strata), and fasting glucose (FG). These markers are integrated into a **composite cardiometabolic risk score (cCMRS)**, representing a continuous endophenotype derived from standardized (z-scored) WHtR, BP, and FG.

A notable analytical feature is the **waist-centered cardiometabolic phenotype**—the clustering of elevated WHtR, BP, and FG in BMI-normal individuals—interpreted as a latent, early risk typology indicating metabolic inflexibility and vascular strain before overt disease.

5. Interpretation Layer

Associations are interpreted as population-level signals, not causal effects, consistent with the cross-sectional design. The model emphasizes longitudinal feedback—subsequent survey waves will validate observed patterns through biomarker calibration (e.g., urinary sodium–potassium ratio) and device-based physical activity monitoring.

6. Programmatic and Policy Utility

Findings are positioned to inform both surveillance and intervention design. Policy levers include salt reduction, fat-quality improvement (e.g., trans-fat bans, PUFA promotion), and strategies targeting ultra-processed foods and sugar-sweetened beverages. The framework aligns with **WHO "Best Buys"** and **UN SDG 3.4** (noncommunicable-disease mortality reduction), establishing a foundation for actionable youth health indicators and evidence-based prevention.

7. Clinical and Epidemiological Utility

The framework's clinical value lies in its **sentinel function**—an early detection and molecular surveillance system capable of identifying subclinical risk constellations before clinical onset. Epidemiologically, it links nutrient exposures to quantifiable metabolic phenotypes, bridging mechanistic insights and public-health surveillance.

Figure S5. NutriSYN framework

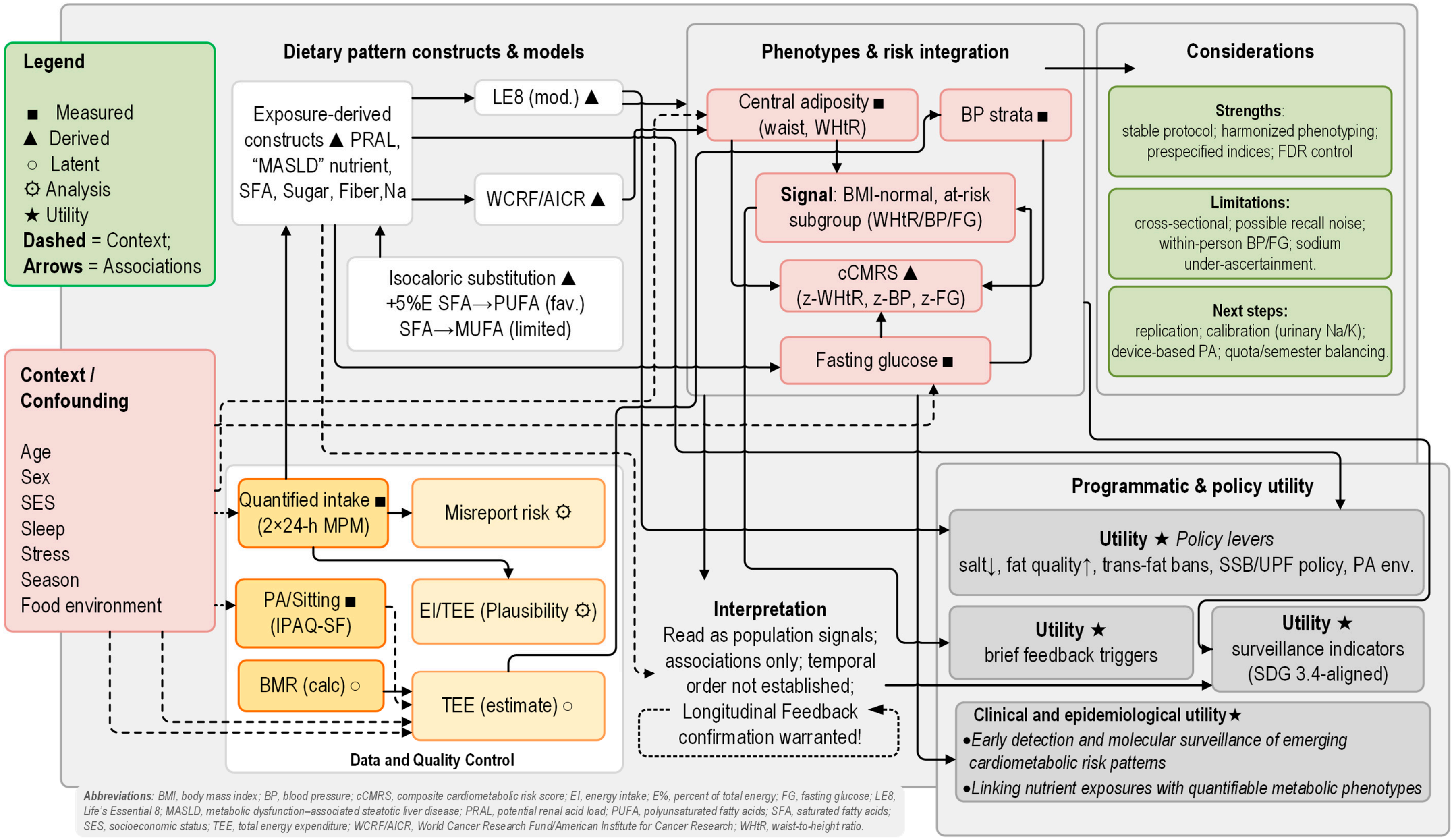

Abbreviations: BMI, body mass index; BP, blood pressure; cCMRS, composite cardiometabolic risk score; EI, energy intake; E%, percent of total energy; FG, fasting glucose; LE8, Life's Essential 8; MASLD, metabolic dysfunction–associated steatotic liver disease; PRAL, potential renal acid load; PUFA, polyunsaturated fatty acids; SFA, saturated fatty acids; SES, socioeconomic status; TEE, total energy expenditure; WCRF/AICR, World Cancer Research Fund/American Institute for Cancer Research; WHtR, waist-to-height ratio.

S1. Overview and Rationale (expanded)

Purpose. Young-adult cardiometabolic (CM) risk is often subclinical and distributed across multiple biological domains (adiposity, vascular tone, glycemic regulation, hepatic metabolism, acid–base balance). A single marker is insufficient; therefore we operationalized complementary, pre-specified indices that map mechanistic pathways to nutrition and activity exposures.

Principles.

1. Comparability: Indices were chosen for uptake across cohorts and alignment with WHO/STROBE-nut reporting (e.g., PRAL, WCRF/AICR, LE8-like constructs).
2. Directionality: Unless defined otherwise by the originating scale, higher = less favorable to simplify interpretation across indices.
3. Energy-aware: Dietary inputs use energy adjustments appropriate to construct (e.g., %E for macronutrients; g/1000 kcal for fiber; mg/day for PRAL minerals).
4. Transparency: All algorithms are explicit; data handling is versioned (database build ID, codebook version, SOP reference).

S2. Data Preparation and Unit Harmonization

Robust surveillance and inference in nutritional epidemiology depend fundamentally on the quality and harmonization of exposure and phenotype data. In this sentinel cohort, data preparation followed a pre-specified, multi-tiered workflow designed to ensure unit consistency, measurement comparability, and traceable provenance across anthropometric, dietary, and metabolic domains.

S2.1. Data Infrastructure and Versioning

All data were entered and managed within a locked dataset version (nutrisyn_wave1_v2.1), maintained under controlled access. Each record carried a unique de-identified participant ID, ensuring linkage across dietary, anthropometric, and biochemical datasets without direct identifiers. Data dictionaries were created for all variables, specifying units, permissible ranges, decimal separators, and missing-value codes. Each transformation (e.g., energy adjustment, standardization, score computation) was scripted in R (v4.5.1) and Python (v3.13.7) to guarantee reproducibility and minimize transcription error. Versioned code repositories were stored in the nutrisyn analytical archive (accessible upon request).

S2.2. Anthropometric and Clinical Data

Measurement Procedures

- Height and weight were measured using calibrated stadiometers and digital scales following WHO STEPS protocols (duplicate readings averaged).
- Waist circumference (WC) was measured midway between the lowest rib and iliac crest; hip circumference (HC) at the maximal gluteal prominence.
- Waist-to-height ratio (whtr) = WC (cm) / height (cm).

- Body mass index (BMI) = weight (kg) / height² (m²).

All devices were recalibrated daily and cross-checked weekly against standardized reference measures.

Blood Pressure

BP was measured twice in the seated position after ≥5 minutes rest using validated oscillometric devices with appropriate cuff sizes.

- The mean of two readings was used.
- Mean arterial pressure (MAP) was derived as:

$$\boldsymbol{MAP = DBP + \frac{(SBP - DBP)}{3}}$$

This continuous composite was used as the vascular component in ccmrs.

Fasting Glycemia

Venous samples were taken after ≥8 h fasting and analyzed on-site using a portable glucometer validated against ISO 15197:2013 standards.

- Units harmonized to mg/dl (1 mmol/L = 18.0182 mg/dl).
- For epidemiologic context only, values were categorized as:
  - Normal: <100 mg/dl
  - Impaired fasting glucose (IFG): 100–125 mg/dl
  - Provisional diabetes: ≥126 mg/dl

No diagnostic inference was made; participants above the provisional threshold were referred to clinical follow-up.

S2.3. Dietary Data

Collection and Entry

Dietary intake was assessed via two non-consecutive 24-hour recalls, interviewer-administered using the Multiple-Pass Method. Interviews were conducted face-to-face under standardized conditions. Data were entered into the nutrisurvey platform, linked to a custom Albanian food-composition database adapted from previous work and harmonized against mccance & Widdowson (Seventh Edition) for nutrient content equivalence.

Portion Size Estimation

A pictorial portion-size atlas, developed ad hoc for the Albanian context, was used to standardize estimation of portion size. Each portion photo corresponded to quantifiable gram weights based on direct measurement of local dishes and household utensils. The atlas included calibration anchors (e.g., standard 100-LEK coin, 200-ml glass).

Quality Control

- Consistency checks: automated verification for decimal placement, missing units, and extreme intakes (≤500 kcal/day or ≥5000 kcal/day).
- Inter-observer reliability: 10% of recalls double-coded by a separate nutritionist; inter-rater correlation coefficient ≥0.92.
- Plausibility checks: energy intake compared to total energy expenditure (TEE) estimates, see Section S9.

S2.4. Harmonization of Nutrient Units

Nutrients were expressed in standardized epidemiologic metrics:

Domain	Metric	Rationale
Macronutrients	% of total energy (%E)	Enables isocaloric substitution modeling
Fiber	G/1000 kcal	Normalizes for energy intake and allows density comparison
Fatty acids	G/1000 kcal	Reflects compositional intake
Minerals & vitamins	Mg/day or µg/day	Absolute intake required for PRAL and adequacy assessment
Sodium, potassium, magnesium, calcium, phosphorus	Mg/day	Required for PRAL equation
Amino acids	G/1000 kcal	Protein quality and acid–base load estimation
Alcohol	Drinks/day (1 drink = 14 g ethanol)	Epidemiologic comparability

S3. Dietary Renal Acid Load (PRAL)

S3.1. Conceptual Basis

The dietary potential renal acid load (PRAL) quantifies the net acid–base effect of nutrient metabolism on systemic acid–base equilibrium. It estimates the difference between acid-generating nutrients (principally sulfur-containing amino acids and phosphorus) and base-generating nutrients (potassium, magnesium, calcium).

From a physiological standpoint, a persistently positive PRAL indicates an acidogenic dietary milieu, which can increase renal acid excretion demands, reduce urinary citrate, and influence long-term risk of hypertension, nephrolithiasis, insulin resistance, and bone resorption. Conversely, a negative or near-neutral PRAL reflects alkali-producing diets, typically richer in fruits, vegetables, and plant-based proteins.

In epidemiologic applications, PRAL serves as a composite exposure marker that integrates multiple nutrient signals into a single acid–base axis, providing a biologically plausible pathway linking diet composition with cardiometabolic outcomes.

S3.2. Computational Formula

PRAL was computed for each participant using the validated Remer–Manz equation (Remer & Manz, Am J Clin Nutr, 1995), expressed in milliequivalents per day (meq/day):

$$\boldsymbol{PRAL} = \mathbf{0.49} \times \text{Protein (g)} + \mathbf{0.037} \times \text{Phosphorus (mg)} - \mathbf{0.021} \times \text{Potassium (mg)} - \mathbf{0.026} \times \text{Magnesium (mg)} - \mathbf{0.013} \times \text{Calcium (mg)}$$

All nutrient inputs were averaged across the two 24-h recalls and expressed in daily units (mg or g as appropriate).

To facilitate comparability, PRAL values were also expressed as energy density (meq/1000 kcal) in sensitivity analyses, though the primary model used absolute PRAL (meq/day) given its physiological relevance to renal acid excretion.

S3.3. Data Inputs and Harmonization

Input nutrients were extracted from the harmonized Albanian food-composition database (see Section S2). Each component underwent quality checks:

- Protein (g/day): sum of animal + plant sources.
- Phosphorus (mg/day): adjusted to include additives in processed foods (where composition data were available).
- Potassium, magnesium, calcium (mg/day): aggregated from all sources, verified against known food composition ranges.

Units were standardized prior to equation application to prevent scaling error. Validation checks ensured that daily PRAL values aligned with plausible population ranges (−100 to +120 meq/day).

S3.4. Analytical Treatment

PRAL was modeled as both:

- Continuous variable (log-transformed when used in regression to normalize right-skewed distribution):

$$\text{Ln(PRAL+100)(offset used to retain negative values)}$$

- Descriptive strata (negative, neutral, positive) in exploratory tables.

For interpretability, regression coefficients were back-transformed and expressed as percent differences per 5%E macronutrient reallocation (see energy-partition modeling in Section S9).

S3.5. Interpretation and Epidemiologic Use

From an epidemiologic perspective, PRAL functions as a metabolic fingerprint of dietary structure rather than a diagnostic biomarker. In this cohort, higher PRAL reflects protein- and phosphate-heavy, mineral-poor diets—patterns characteristic of Westernized, processed food consumption.

Tracking PRAL distributions longitudinally provides insight into shifts in population-level dietary quality and can serve as a sensitive early indicator for policy impact (e.g., reformulation, fruit–vegetable intake programs).

PRAL's inclusion in the sentinel system thus satisfies three criteria:

1. Feasibility — requires only standard nutrient inputs.
2. Mechanistic interpretability — directly linked to acid–base physiology and renal strain.
3. Comparability — widely used internationally in population surveys and interventional studies.

S3.6. Validation and Sensitivity Checks

- Internal validation: PRAL values were cross-checked against estimated potassium-to-protein ratios (inverse correlation expected, r ≈ −0.65 in comparable cohorts).
- Distributional sanity checks: Histogram inspection confirmed expected right-skew with modal range +10 to +30 meq/day.
- Sex differences: As anticipated, men exhibited higher PRAL (due to higher protein and phosphorus intakes and lower fruit/vegetable consumption).
- Energy-density correction sensitivity: When expressed per 1000 kcal, correlation with absolute PRAL remained >0.9, confirming proportionality and stability of the index.

S3.7. Integration with Other Indices

Within the broader analytic framework:

- PRAL was used as one of the primary outcomes in substitution models to test diet-quality effects (e.g., SFA → PUFA reallocation).
- It was cross-referenced with MASLD nutrient score (Section S4) to explore hepatic–renal nutritional coupling, a concept increasingly recognized in systems epidemiology.
- PRAL and MASLD together formed the FDR-controlled primary family for multiplicity correction ($q < 0.05$).

S3.8. Strengths and Limitations of PRAL Application

Strengths: mechanistically grounded, easy to compute, sensitive to real-world dietary shifts, interpretable by both clinicians and policymakers.

Limitations: does not capture non-dietary acid load contributors (e.g., renal function, medication), assumes average nutrient absorption, and

relies on recall-based nutrient data. Nevertheless, in a surveillance context, it remains one of the most robust nutrient-derived composite indices for acid–base and renal-metabolic stress.

Summary:

PRAL offers a mechanistically coherent, empirically validated, and low-cost lens into diet-related renal and metabolic load. Its inclusion anchors the sentinel surveillance system in quantitative physiology, bridging nutrient-level exposures with early subclinical phenotypes.

S4. MASLD-Oriented Nutrient Score

S4.1. Conceptual Rationale

The MASLD-oriented nutrient score (Metabolic dysfunction–Associated Steatotic Liver Disease) is a nutrient-based composite proxy designed to approximate the dietary contribution to hepatic lipid accumulation, oxidative stress, and metabolic dysfunction.

MASLD (formerly NAFLD) reflects the hepatic manifestation of systemic metabolic imbalance and is tightly linked to diet composition — particularly fat quality, fructose and free sugar exposure, micronutrient insufficiency, and pro-inflammatory nutrient excess.

In population surveillance, clinical imaging or biopsy-based MASLD diagnosis is not feasible. Thus, a nutrient-oriented score provides a scalable, surveillance-compatible surrogate that captures the dietary patterns mechanistically associated with hepatic steatosis risk.

This index follows the principle of etiologic fidelity under pragmatic measurement — that is, maintaining alignment with mechanistic pathways even when operationalized through population-level data.

S4.2. Construct and Component Selection

The MASLD-oriented nutrient score was pre-specified a priori, built from nutrients with reproducible links to hepatic steatosis, insulin resistance, and oxidative stress, informed by meta-analyses and controlled feeding trials.

The following nutrient groups were incorporated, each oriented such that higher values reflect a less favorable hepatic–metabolic signature:

Domain	Included Nutrients	Directionality (↑ = less favorable)	Rationale
Lipids	Saturated fatty acids (SFA, %E), cholesterol (mg/1000 kcal)	↑	Promote hepatic lipogenesis and ER stress
Carbohydrates	Free sugars (%E), total fructose (g/1000 kcal)	↑	Drive de novo lipogenesis and hepatic fat storage

Protein	Animal protein (g/1000 kcal)	↑	Reflects methionine/sulfur amino acid burden contributing to oxidative load
Micronutrients (Protective)	Vitamin E (mg/1000 kcal), vitamin D (μg/day), folate (μg/day), magnesium (mg/day), potassium (mg/day), fiber (g/1000 kcal)	↓ (inverse score)	Anti-inflammatory, antioxidant, insulin-sensitizing roles
Fat quality (Protective)	Polyunsaturated fatty acids (PUFA, %E), PUFA/SFA ratio	↓ (inverse score)	Enhance hepatic β-oxidation and reduce triglyceride synthesis

S5. WCRF/AICR Adherence Score

S5.1. Conceptual Foundation

The World Cancer Research Fund/American Institute for Cancer Research (WCRF/AICR) 2018 adherence score operationalizes compliance with global evidence-based recommendations for diet, nutrition, and physical activity in cancer prevention.

Although originally conceived for cancer outcomes, these recommendations encompass lifestyle factors (energy balance, diet composition, and body composition) that also underpin cardiometabolic health and metabolic-dysfunction–associated conditions, making the index suitable for integrated NCD surveillance.

The use of this score in the present study extends its utility to a younger adult population, emphasizing the shared preventive pathways linking diet, obesity, and metabolic dysfunction across the life course — a perspective increasingly endorsed by IARC, WHO/Europe, and global NCD frameworks.

S5.2. Index Structure and Scoring Logic

We applied the 2018 WCRF/AICR "Third Expert Report" scoring framework (Shams-White et al., Nutrients, 2019), adapted to the Albanian setting, with seven applicable components. Each component was scored as 1 (fully met), 0.5 (partially met), or 0 (not met), based on quantitative cut-points defined by the WCRF/AICR Cancer Prevention Recommendations.

Component	Criterion (Simplified for Surveillance)	Scoring Approach	Epidemiologic Rationale
1. Healthy body weight	BMI between 18.5–24.9 kg/m²	1 = within range, 0.5 = marginal deviation, 0 = outside range	Obesity is a shared risk factor for cancer and cmds
2. Physical activity	≥150 min/week moderate or ≥75 min/week vigorous PA	1 = meets, 0.5 = near-miss (≥120 min), 0 = below	Energy balance and insulin sensitivity
3. Diet rich in whole grains, fruits, vegetables, and beans	≥5 portions/day fruits/vegetables and >50% whole grains	1/0.5/0	Plant-forward patterns reduce inflammation and oxidative stress
4. Limit fast/ultra-processed energy-dense foods	<20% of total energy from upfs (proxy via SFA + added sugars + low fiber)	1/0.5/0	Reflects overall dietary processing level
5. Limit red and processed meat	<350–500 g/week red meat, <50 g/week processed	1/0.5/0	Saturated fat, nitrates, and heme iron pathways
6. Limit sugar-sweetened beverages (ssbs)	<1 serving/week (250 ml)	1/0.5/0	Added sugars, insulin resistance, hepatic fat
7. Limit alcohol consumption	0 drinks/day (abstainer)	1 = 0 drinks, 0.5 = ≤1 drink/day, 0 = >1 drink/day	Carcinogenic and metabolic effects

S6. Modified Life's Essential 8 (LE8) Score

S6.1. Conceptual Framework

The Life's Essential 8 (LE8) is a composite construct developed by the American Heart Association (AHA, 2022) to operationalize cardiovascular health as a multidimensional state integrating lifestyle behaviors and health factors. In its canonical form, LE8 encompasses eight metrics—diet, physical activity, nicotine exposure, sleep, BMI, blood pressure, lipids, and glucose—each scored on a 0–100 scale. Higher total scores represent better overall cardiovascular health (CVH).

Given the surveillance scope and pragmatic constraints of the current study, a modified six-component version was implemented, encompassing available, high-fidelity domains:

1. Diet
2. Physical activity
3. Body mass index (BMI)

4. Blood pressure (BP)
5. Fasting glucose (FG)
6. Smoking status (as a proxy for nicotine exposure)

Sleep and laboratory lipids were unavailable in this wave and therefore omitted, with acknowledgment that these will be incorporated in subsequent waves as the sentinel platform expands to biochemical measures.

The adaptation adheres to the principle of informative minimalism—preserving core construct validity while ensuring feasibility and reproducibility in low-resource epidemiologic contexts.

S6.2. Scoring Approach and Computational Method

Each metric was transformed onto a 0–100 scale, following AHA algorithms and international reference cut-points. Component scores were averaged (simple mean, equal weighting) to produce the modified LE8 total score (0–100).

Domain	Input Variable(s)	0 (Low CVH)	50 (Intermediate CVH)	100 (High CVH)	Data Source
Diet	WCRF-based diet-quality composite	<3/7	3–5/7	≥6/7	24-h recalls
Physical Activity	IPAQ-SF total MET-min/week	<600	600–1200	≥1200	IPAQ-SF
BMI (kg/m²)	Measured	≥30	25–29.9	18.5–24.9	Anthropometry
Blood Pressure (mmhg)	Mean of two readings	≥130/85	120–129/80–84	<120/<80	Seated BP
Fasting Glucose (mg/dl)	Measured	≥100	90–99	<90	Capillary glucose
Smoking Status	Self-report	Current	Former	Never	Questionnaire

S7. Composite Cardiometabolic Risk Score (ccmrs)

S7.1. Rationale and Conceptual Framework

The Composite Cardiometabolic Risk Score (ccmrs) was designed as a continuous, standardized metric that quantifies the clustering of early cardiometabolic perturbations across three physiologic domains: adiposity, vascular function, and glucose regulation.

Its conceptual basis derives from the principle that cardiometabolic risk evolves gradually, beginning as subclinical shifts in body composition, hemodynamics, and glucose homeostasis long before overt disease emerges.

While categorical thresholds (e.g., hypertension, impaired fasting glucose, obesity) are clinically convenient, they are statistically inefficient in small or homogeneous samples and fail to capture the continuum of metabolic risk. The ccmrs circumvents this limitation by integrating multiple continuous markers into a single z-standardized construct, sensitive to early co-occurrence and progression.
This approach aligns with WHO's stepwise surveillance philosophy and principle of dimensional epidemiology—detecting gradients, not just events.

S7.2. Components and Calculation

The ccmrs incorporates three core biomarkers, chosen for their reproducibility, accessibility, and pathophysiologic complementarity:

Domain	Indicator	Unit	Interpretation
Adiposity	Waist-to-height ratio (whtr)	Unitless	Central fat distribution; anthropometric surrogate of visceral adiposity
Vascular	Mean arterial pressure (MAP) = DBP + (SBP–DBP)/3	Mmhg	Integrative indicator of vascular tone and load
Glycemic	Fasting glucose (FG)	Mg/dl	Reflects insulin sensitivity and hepatic glucose output

S8. Data Quality Control and Reliability Framework

S8.1. Rationale and Governance

High-quality surveillance data require consistency, traceability, and internal validity equivalent to clinical research standards.

Given that Albania lacks pre-existing nutrition-epidemiologic infrastructure, the nutrisyn sentinel framework was explicitly designed to establish methodological sovereignty—ensuring that all core data domains (anthropometry, diet, physical activity, biochemical proxies) are reproducible and auditable over time.

All procedures followed WHO STEPS and STROBE-Nut methodological guidelines, overseen by a central data-quality board within the coordinating institution.
This section documents the full data-assurance workflow, from participant screening to final analytic dataset generation.

S8.2. Quality-Assurance Architecture

Quality assurance (QA) was built into every operational layer:

Level	Objective	Key Procedures	Verification Frequency
1. Field operations	Standardize participant assessment	SOP training, inter-observer calibration, duplicate readings	Daily
2. Data capture	Ensure accurate entry and unit harmonization	Double data entry, range/logic checks, real-time digital prompts	Continuous

3. Central data audit	Validate completeness and plausibility	Random 10% audit, reconciliation log	Weekly
4. Statistical QA	Detect drift, implausible outliers, or systemic bias	Distributional QC plots, duplicate record check, sex-specific sanity checks	Post-collection
5. Documentation	Guarantee reproducibility and transparency	Codebook version control, variable lineage map, SOP repository	Continuous

S9. Statistical Modeling Framework

S9.1. Analytical Philosophy

The modeling framework was designed to quantify diet–phenotype associations under a surveillance paradigm—emphasizing directional insight and reproducibility, not causal inference.

The analytical structure followed three methodological imperatives:

1. Physiologic coherence – models mirror metabolic substitution (e.g., SFA → PUFA exchange).
2. Statistical sufficiency – pre-specified covariates defined a minimal sufficient adjustment set derived from causal diagrams (dags) reflecting shared determinants (age, sex, smoking, physical activity).
3. Transparency and stability – analytic flexibility was minimized by pre-registering outcome families, modeling forms, and multiple-comparison procedures.

This aligns with principle of aetiologic humility with technical exactness: using sophisticated models without overstating their inference scope.

S9.2. Model Type: Energy-Partition Substitution Framework

To investigate nutrient composition effects independently of total energy intake, analyses employed energy-partition models, a standard in nutritional epidemiology for isolating substitution effects.

Each model estimated the association of increasing one macronutrient's energy contribution at the expense of another (here, carbohydrate), while total energy and other macronutrients were held constant.

$$\boldsymbol{Y = \beta_0 + \beta_1(E_{SFA}) + \beta_2(E_{PUFA}) + \beta_3(E_{MUFA}) + \beta_4(E_{Protein}) + \beta_5(E_{Alcohol}) + \beta_6(E_{Total}) + C + \epsilon}$$

Where:

- $\boldsymbol{Y}$= outcome (e.g., PRAL, MASLD nutrient score, ccmrs, LE8, WCRF, SBP, FG, whtr)
- $\boldsymbol{E_i}$= percent energy from macronutrient i
- $\boldsymbol{C}$= vector of covariates (age, sex, smoking status, physical activity [continuous IPAQ total mets])
- $\boldsymbol{\epsilon}$= residual error term

The sum of macronutrients approximates 100% of energy intake; hence, by omitting one term (carbohydrate), each β coefficient estimates the expected change in outcome per +1%E increase in the nutrient, replacing carbohydrate energy.

This design respects isocaloric substitution logic, ensuring interpretability as a nutrient reallocation rather than a simple intake–outcome correlation.

S9.3. Pre-Specified Substitution Contrasts

The main interest lay in quality of dietary fat, thus two contrasts were defined a priori:

1. SFA → PUFA substitution: $\boldsymbol{\Delta_{PUFA-SFA}} = (\boldsymbol{\beta_{PUFA}} - \boldsymbol{\beta_{SFA}}) \times \mathbf{5}$
2. SFA → MUFA substitution: $\boldsymbol{\Delta_{MUFA-SFA}} = (\boldsymbol{\beta_{MUFA}} - \boldsymbol{\beta_{SFA}}) \times \mathbf{5}$

Multiplying by 5 expresses the difference per +5%E nutrient reallocation, a standard epidemiologic scale corresponding to realistic dietary shifts.

These contrasts quantify directional nutrient-pattern effects on physiologically relevant indices (renal acid load, hepatic nutrient profile, BP, glycemia), providing policy-usable estimates for preventive nutrition.

S9.4. Outcome Families and Multiple-Comparison Control

To safeguard interpretive reliability under multiple correlated outcomes, outcomes were grouped into two pre-specified families:

Family	Primary Outcomes (FDR-controlled)	Secondary Outcomes (Exploratory)
1. Metabolic-signature family	PRAL, MASLD nutrient score	LE8, WCRF adherence
2. Clinical-signature family	SBP, FG, whtr, ccmrs	Individual BP strata

S10. Longitudinal Expansion and Future Calibration Plan

S10.1. Vision and Strategic Rationale

The nutrisyn sentinel platform was conceived not as a one-off cross-sectional survey, but as the foundation of a longitudinal, WHO-aligned surveillance system capable of detecting and validating cardiometabolic (CM) risk trajectories among Albanian youth.

In low- and middle-income settings where structured cohort infrastructures are rare, this approach bridges two scientific imperatives:

1. Surveillance fidelity – tracking exposures and early risk clustering over time.
2. Etiologic resolution – progressively refining causal inference through biomarker calibration and repeated measures.

By maintaining harmonized protocols and periodic follow-up waves, nutrisyn transforms surveillance data into time-anchored population signals, suitable for national policy evaluation and international comparison within WHO/Europe's NCD monitoring framework.

S10.2. Follow-up Architecture

Design

Follow-up is structured in successive 3-year waves (Wave 2 in 2027, Wave 3 in 2030), with the current cohort forming the longitudinal nucleus.

Each wave repeats the full core module (anthropometry, BP, fasting glucose, 24-h dietary recall, IPAQ-SF), while adding higher-resolution modalities to improve measurement precision and aetiologic inference.

Retention and Tracking

Participant retention is supported through:

- Institutional affiliation (universities maintain updated contact rosters),
- Annual digital check-ins via encrypted platforms, and
- Incentivized on-site visits for re-examination (certified health report provided to each participant).

Projected follow-up retention: ≥75% at 3 years, based on pilot response rates and engagement metrics.

S10.3. Planned Calibration Enhancements

Each subsequent wave will incorporate biochemical and objective calibration modules to enhance comparability and validity across exposure and outcome domains.

To correct self-reported dietary and lifestyle data, laboratory markers will be integrated:

Calibration Domain	Planned Biomarker(s)	Rationale
Sodium/Potassium Intake	Spot urine Na/K ratio	Objective validation of dietary salt exposure and PRAL estimation
Glycemic Regulation	Hba1c, fasting insulin	Capture chronic glycemia and early insulin resistance
Lipid Metabolism	Triglycerides, HDL-C, LDL-C	Expand CM profiling beyond BP/FG; enable ccmrs+
Hepatic Function	ALT, GGT	Validate MASLD nutrient score against biochemical hepatic stress markers

Micronutrient Status	25(OH)D, serum folate, ferritin	Validate reported shortfalls in D, folate, and iron intake

S11. Limitations, Replicability, and Methodological Safeguards

S11.1. Overview

All surveillance systems operate under constraints of design, resources, and logistics.
In the case of nutrisyn, these limitations are neither hidden nor exceptional; they are clearly specified, quantifiable, and, crucially, correctable over time.

Rather than diminishing interpretability, such transparency strengthens the study's scientific credibility and its alignment with principles of traceable inference and measurable uncertainty.

S11.2. Study Design and Generalizability

Nutrisyn Wave 1 was cross-sectional and sentinel by design, not population-representative.

Participants were university-enrolled young adults, which positions the study within a defined, analytically stable stratum of the Albanian population rather than as a national prevalence estimate.

This sentinel structure emphasizes trend detection and reproducibility, not extrapolation.

While representativeness across rural or non-student groups is limited, the current cohort provides a crucial reference axis for subsequent, broader surveillance expansions.

Safeguard:

- Clear acknowledgment in all outputs that estimates represent a baseline signal, not national prevalence.
- Future waves will apply stratified sentinel sampling to incorporate technical and vocational populations, enabling calibration of representativeness.

S11.3. Sample Size and Power

The analytic sample (N = 262; 172 women, 90 men) provides adequate power to detect moderate standardized effect sizes (Cohen's d ≈ 0.35) for continuous outcomes under $\alpha = 0.05$ with robust ses. However, the precision for subgroup contrasts and non-linear dose–response modeling remains limited.

Safeguard:

- Effect estimates are expressed with 95% confidence intervals and false-discovery-rate control, prioritizing directionality and reproducibility over statistical significance.
- Future longitudinal waves will pool data across time to enhance statistical power through repeated measures.

S11.4. Cross-Sectional Constraints and Temporality

As a baseline assessment, the cross-sectional design cannot infer temporality or causality. Associations between diet, physical activity, and cardiometabolic markers should therefore be interpreted as co-occurring patterns, not predictive pathways.
However, the primary purpose of nutrisyn Wave 1 is to generate surveillance hypotheses and calibrate operational feasibility for future longitudinal follow-up (Wave 2 onwards).

Safeguard:

- Prospective replication and repeated measurements every three years (see S10) will enable temporal inference once trajectories are established.

S11.5. Dietary Assessment and Recall Bias

Two interviewer-administered 24-hour recalls provide valid group-level estimates but are subject to within-person variability and recall bias. Self-reported diet is also sensitive to social desirability effects, particularly among young adults, where underreporting of energy-dense foods is common.
Given the small stratum size and cultural dietary heterogeneity, absolute nutrient-intake estimates should be interpreted as approximations.

Safeguard:

- Harmonized multiple-pass protocol with trained nutrition graduates and culturally adapted portion-size atlas minimizes systematic bias.
- Implausible reporters were identified using Goldberg cut-offs (TEI:TEE ratio) but not excluded, preserving comparability and avoiding selective trimming bias.
- Future biomarker calibration (e.g., urinary sodium, 24-h nitrogen balance, stable isotope validation) will permit correction for misreporting.

S11.6. Energy Partition and Substitution Modeling

Isocaloric substitution models assume stable total energy and no unmeasured confounding between macronutrients — assumptions that may be partially violated in free-living samples. Given the cross-sectional nature and modest sample size, such models should be interpreted as directional indicators of nutrient quality rather than as effect estimators.

Safeguard:

- Models were pre-specified and adjusted a priori for sex, age, smoking, and physical activity.
- Benjamini–Hochberg false-discovery-rate control ($q < 0.05$) was applied to reduce multiplicity bias.
- The substitution approach remains conceptually sound for exploratory public-health surveillance and for diet–phenotype mapping.

S11.7. Measurement Error in Clinical Markers

BP and FG were measured under standardized, fasting conditions but remain susceptible to short-term variation (hydration, stress, diurnal rhythm). The single-occasion fasting glucose used here cannot replace diagnostic confirmation of diabetes. Thus, terms such as impaired fasting glucose or provisional diabetes are descriptive epidemiologic categories, not clinical diagnoses.

Safeguard:

- Duplicate BP readings and standardized timing reduce random error.
- Future waves will incorporate duplicate fasting measures, hba1c, and insulin assays for improved reliability and homeostatic-modeling precision.

S11.8. Residual Confounding and Covariate Adjustment

Residual confounding is possible given unmeasured variables such as sleep, stress, and genetic predisposition. However, directed acyclic graphs (dags) were used to define minimal sufficient adjustment sets, reducing model overfitting and maintaining interpretability.

Safeguard:

- Robust (HC3) standard errors mitigate heteroskedasticity.
- Future calibration studies will allow incorporation of objective markers (e.g., accelerometry, metabolomics) into confounding structures.

S11.9. Replicability and Analytical Transparency

The analytical workflow was fully documented and scripted in R, Python, and SPSS to ensure reproducibility.
All data cleaning, modeling, and visualization scripts are archived with embedded metadata and will be made publicly accessible post-publication (see S11 and S12).
Safeguard:

- Version-controlled repositories (Git-based) store every analytic step, with checksum verification.
- Harmonized data dictionaries ensure inter-wave comparability and re-analysis feasibility.
- Analytical decisions were pre-specified and logged (e.g., choice of model families, covariates, transformations) to avoid post hoc flexibility.

S11.10. Interpretive Boundaries and Surveillance Role

The observed associations reflect signal detection within a surveillance framework, not etiologic inference. The goal is not to identify causality but to establish a reproducible baseline for Albania's youth metabolic landscape, capable of supporting WHO-aligned early-warning systems.

Safeguard:

- Interpretation is explicitly labeled as surveillance-level in Abstract, Discussion, and Conclusion.
- Language conforms to peer-reviewed editorial standards ("consistent with," "suggestive of," "not causal").

S11.11. Strengths as Inverse of Limitations

When systematically addressed, each limitation translates into a design strength:

- Cross-sectional → reproducible sentinel baseline
- Modest sample size → tractable for calibration and repeated measures
- Self-report → correctable with biomarkers
- Restricted population → analytically homogeneous for mechanistic interpretation

This transparency enables progressive precision: every subsequent nutrisyn wave will reduce bias variance and strengthen the reliability of trend detection.

S11.12. Summary

In sum, nutrisyn's limitations are not liabilities but structural realities managed through explicit safeguards. By adhering to reproducible design principles, rigorous analytic transparency, and progressive calibration, the platform embodies IARC's doctrine of cumulative evidence through accountable surveillance. The cross-sectional baseline represents not an endpoint, but a launchpad for longitudinal validity, where replication is not redundancy—it is scientific discipline.

S12. Integration with Policy Translation and Health-System Uptake

S12.1. Translational Rationale

Nutrisyn was conceived not merely as an academic study but as a functional surveillance prototype for health-policy translation in Albania and, by extension, in similarly transitioning Southeastern European contexts. Its methodological rigor—standardized 24-hour recalls, harmonized anthropometry, and WHO-aligned cardiometabolic phenotyping—positions it as an early-warning instrument for

cardiometabolic risk detection among young adults. By quantifying modifiable exposures (e.g., dietary fat quality, sodium load, physical inactivity) and their co-occurrence with early risk markers (e.g., central adiposity, elevated BP, impaired glycemia), nutrisyn creates an evidence feedback loop linking population data to prevention policy. This alignment satisfies core translational principle: that epidemiology must ultimately serve decision-making and accountability, not remain confined to inference.

S12.2. Linkage to WHO and EU Frameworks

Nutrisyn's data structure and indicators are directly mappable to global and regional monitoring frameworks, including:

Policy Framework	Relevant nutrisyn Indicator	Mechanism of Integration
WHO NCD "Best Buys"	Salt intake, fat quality, sugar intake, physical activity	Integration into WHO-Europe country dashboards
EU DG-SANTE Core Health Indicators	BMI, BP, diet quality, PA, tobacco use	Periodic reporting within EU accession progress
WHO STEPS 2.0	Harmonized BP, anthropometry, physical activity modules	Direct template compatibility
SDG 3.4 and 3.d Targets	Premature NCD mortality, surveillance capacity	Basis for early prevention monitoring
EHII / European Health Data Space (EHDS)	Nutrient patterns, behavioral determinants	Data interoperability for EU-level analyses

S13. Communication, Dissemination, and Public Engagement

S13.1. Translational Philosophy

Nutrisyn's communication strategy is built on a simple premise:

Science must circulate to be useful. Data that remain confined to academic circles cannot improve population health. Accordingly, nutrisyn follows the IARC and WHO-Europe standards of transparent, non-sensational dissemination, combining the clinical precision of epidemiology with the clarity required for broad public understanding. Its public-health communication is guided by three principles:

1. Accuracy over alarmism: All messages derive directly from peer-reviewed findings, avoiding moral or behavioral judgment.
2. Comprehensibility: Data are expressed in accessible terms (e.g., "one in three young adults has early signs of metabolic risk") while preserving nuance.
3. Actionability: Each message is linked to a modifiable behavior or policy lever—salt, fat, sugar, or movement—making the information usable by both citizens and decision-makers.

S13.2. Communication Architecture

Nutrisyn's dissemination framework operates across three concentric circles:

1. Scientific Community: Peer-reviewed papers, conference presentations, and open-access data repositories.

2. Policy Makers and Health Institutions: Briefing notes, dashboards, and visual policy memos tailored to ministries, WHO offices, and EU partners.
3. General Public and Media: Simplified infographics, press statements, and digital narratives co-developed with communication specialists.

Each layer reinforces the others—ensuring that translation occurs without distortion.

S13.3. Core Communication Products

1. Policy Briefs and Executive Summaries

Concise, data-rich briefs summarizing key indicators (BMI, BP, sodium intake, diet quality, activity levels) are distributed to ministries and parliamentary committees. Each brief includes:

- The problem (e.g., “63% of male students have elevated blood pressure despite normal BMI”),
- The opportunity (“salt reduction and improved fat quality can reduce risk within one year”), and
- The action (“enforce WHO-aligned sodium reformulation and youth screening”).

2. Annual Sentinel Report

An open-access annual report visualizes key indicators, trend trajectories, and regional comparisons with WHO/Europe and EU averages. This report uses Nature Medicine–style figure design: clear benchmarks, transparent legends, and short interpretive captions (in English and Albanian).

3. Media Engagement Toolkit

Includes:

- Pre-approved scientific quotes (for TV/radio journalists);
- Visual assets (charts, icons, EU-compliant color palettes);
- Short “explainer” videos (30–60 seconds) highlighting findings without oversimplification;
- Guidelines for responsible coverage (avoiding “obesity blame” narratives).

4. Public-Facing Infographics

Produced in collaboration with design partners and WHO communication officers, these use human-centered framing (e.g., “Every third student already shows early signs of high blood pressure”) rather than abstract percentages. Each infographic pairs a risk message with a feasible behavioral alternative (“add potassium-rich foods instead of cutting entire meals”).

S14. Integration of the University of Medicine and Other National Institutions: Expanding Expertise, Scope, and Collaboration in Albania

S14.1. Strategic Imperative for Institutional Integration

The nutrisyn sentinel system was conceived as a national scientific commons—a platform to generate, calibrate, and share data on diet and cardiometabolic health in Albanian youth.

Its long-term viability depends on anchoring it within Albania's existing academic and public-health institutions, most notably the University of Medicine, Tirana (UMT), Agricultural University of Tirana (AUT), and National Institute of Public Health (NIPH).

Institutional integration ensures three core outcomes:

1. Expertise continuity – safeguarding methodological quality through academic stewardship.
2. Data sustainability – embedding surveillance within national structures that outlive individual projects.
3. Policy translation – linking evidence generation with ministries and decision-makers.

S14.2. University of Medicine, Tirana (UMT): Clinical and Epidemiologic Nexus

UMT offers clinical infrastructure, laboratory facilities, and domain expertise in internal medicine, endocrinology, and epidemiology—resources essential for expanding nutrisyn's scope from anthropometric and dietary surveillance to biomarker validation and early-disease phenotyping.

Planned integration will include:

- Joint epidemiology and biostatistics unit with shared staff appointments between AUT and UMT, ensuring consistent quality control and methodological training.
- Clinical interface for participants identified with abnormal BP or glucose values, enabling standardized referral and longitudinal follow-up under ethical oversight.
- Shared biomarker laboratory protocols for fasting glucose, lipid panels, and emerging molecular markers (e.g., hba1c, ALT, GGT), harmonized under ISO 15189 standards.
- Research training modules for medical and nutrition students on surveillance analytics, fostering a new cadre of epidemiologists skilled in real-world data integration.

Through this collaboration, UMT becomes the clinical spine of nutrisyn—providing diagnostic and translational depth while the sentinel platform retains its independent epidemiologic governance.

S14.3. Agricultural University of Tirana (AUT): Nutritional Epidemiology Core

AUT remains the foundational host institution and methodologic custodian of nutrisyn.

Its role includes:

- Coordinating dietary-recall standardization and maintenance of the Albanian Food Composition Database.
- Conducting calibration studies and portion-size validation using the AUT nutrition laboratories.
- Hosting the nutrisyn Data Repository, ensuring secure storage and controlled access to anonymized datasets.

- Training field assessors and maintaining continuous quality-assurance audits of data collection.

This ensures that the platform's scientific and technical standards remain consistent with STROBE-Nut, WHO STEPS, and IARC's nutritional surveillance principles.

S14.4. National Institute of Public Health (NIPH): Surveillance and Policy Interface

The NIPH will serve as the national policy conduit for nutrisyn outputs. Integration will occur through:

- Formal data-sharing agreements allowing nutrisyn indicators to feed directly into NCD dashboards and WHO-Europe reporting.
- Joint methodological workshops on data harmonization, quality assurance, and cross-survey comparability (linking nutrisyn with WHO STEPS and DHS datasets).
- Policy briefs and situation reports co-signed by NIPH, ensuring institutional legitimacy and policy uptake of surveillance findings.
  NIPH's participation transforms nutrisyn from an academic initiative into an operational component of Albania's NCD surveillance infrastructure.

S14.5. Ministry of Health and Social Protection: Policy Ownership

For sustainability, the Ministry's role will be to institutionalize nutrisyn as a recognized national surveillance pillar under the upcoming Albanian NCD Action Plan 2030.

Key actions include:

- Embedding nutrisyn indicators (e.g., sodium intake, fat-quality ratio, PRAL, MASLD nutrient score) into national monitoring frameworks.
- Supporting policy translation through fiscal and regulatory levers (SSB tax reinstatement, reformulation, youth screening).
- Financing core operational costs under the public-health surveillance budget line, ensuring long-term continuity.

S14.6. University Network Expansion

Beyond Tirana, nutrisyn will engage regional universities—Shkodër, Korçë, Elbasan, and Vlora—as sentinel satellite centers to enhance geographical coverage and equity. Each site will replicate the standardized protocol, contributing to pooled analyses and regional disaggregation. Data harmonization will be assured through:

- Centralized digital templates for 24-h recalls, anthropometry, and BP measurement;
- Cross-site inter-observer calibration studies every two years;
- Cloud-based version control for all instruments and variable dictionaries.
  This creates a distributed network of competence capable of supporting future WHO-aligned surveillance or EU Joint Action participation.

S14.7. Multidisciplinary and Cross-Sectoral Partnerships

To strengthen domain breadth, nutrisyn will seek collaboration with:

- Faculty of Natural Sciences (University of Tirana): biochemistry and metabolomics integration;
- Institute of Statistics (INSTAT): sampling, representativeness, and national extrapolation;
- Institute of Public Health Nutrition Laboratory: biochemical marker cross-validation;
- Civil Society and ngos: community engagement and youth awareness;
- Private Sector (regulated): data co-creation in reformulation and labeling compliance.

All partnerships will be governed by transparent Memoranda of Understanding specifying data ownership, authorship criteria, and conflict-of-interest safeguards.

S14.8. Summary

The integration of the University of Medicine, Agricultural University, and public-health institutions marks a pivotal shift—from a single-site academic initiative to a nationally embedded surveillance ecosystem. Through shared expertise, harmonized standards, and coordinated governance, Albania can consolidate a sustainable platform for youth cardiometabolic monitoring that is scientifically credible, institutionally legitimate, and internationally interoperable. This model—linking academia, health services, and government—sets the precedent for small countries seeking to operationalize WHO-aligned nutrition surveillance with limited resources but high scientific integrity.
